# Advancements in nanohydroxyapatite: synthesis, biomedical applications and composite developments

**DOI:** 10.1093/rb/rbae129

**Published:** 2024-11-05

**Authors:** Rui Zhao, Xiang Meng, Zixian Pan, Yongjia Li, Hui Qian, Xiangdong Zhu, Xiao Yang, Xingdong Zhang

**Affiliations:** School of Medicine, Department of Inspection, Jiangsu University, Zhenjiang 212013, China; School of Medicine, Department of Inspection, Jiangsu University, Zhenjiang 212013, China; School of Medicine, Department of Inspection, Jiangsu University, Zhenjiang 212013, China; School of Medicine, Department of Inspection, Jiangsu University, Zhenjiang 212013, China; School of Medicine, Department of Inspection, Jiangsu University, Zhenjiang 212013, China; National Engineering Research Center for Biomaterials, Sichuan University, Chengdu 610064, China; National Engineering Research Center for Biomaterials, Sichuan University, Chengdu 610064, China; National Engineering Research Center for Biomaterials, Sichuan University, Chengdu 610064, China

**Keywords:** nanohydroxyapatite, composite material, bone regeneration, dentistry, cancer therapy

## Abstract

Nanohydroxyapatite (nHA) is distinguished by its exceptional biocompatibility, bioactivity and biodegradability, qualities attributed to its similarity to the mineral component of human bone. This review discusses the synthesis techniques of nHA, highlighting how these methods shape its physicochemical attributes and, in turn, its utility in biomedical applications. The versatility of nHA is further enhanced by doping with biologically significant ions like magnesium or zinc, which can improve its bioactivity and confer therapeutic properties. Notably, nHA-based composites, incorporating metal, polymeric and bioceramic scaffolds, exhibit enhanced osteoconductivity and osteoinductivity. In orthopedic field, nHA and its composites serve effectively as bone graft substitutes, showing exceptional osteointegration and vascularization capabilities. In dentistry, these materials contribute to enamel remineralization, mitigate tooth sensitivity and are employed in surface modification of dental implants. For cancer therapy, nHA composites offer a promising strategy to inhibit tumor growth while sparing healthy tissues. Furthermore, nHA-based composites are emerging as sophisticated platforms with high surface ratio for the delivery of drugs and bioactive substances, gradually releasing therapeutic agents for progressive treatment benefits. Overall, this review delineates the synthesis, modifications and applications of nHA in various biomedical fields, shed light on the future advancements in biomaterials research.

## Graphical abstract

**Figure:**
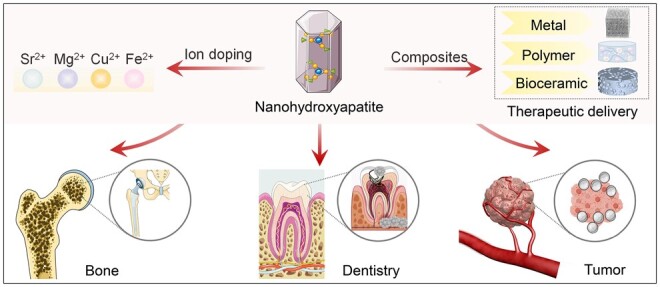

## Introduction

Synthetic nanohydroxyapatite [nHA, Ca_10_(PO_4_)_6_(OH)_2_] has attracted extensive attention in biomedical research due to its biocompatibility, high bioactivity and osteoconductive properties [1]. The biological functionality of nHA is intricately linked to its physical characteristics, including morphology, aspect ratio, crystallinity and concentration [2, 3]. In bone tissue engineering, the implantation of synthetic nHA into bone defects initiates new bone formation and bonding to the material, which is crucial for the reconstruction of damaged bone tissue [4, 5]. This osteogenic potential is primarily attributed to nHA’s ability to induce the differentiation of mesenchymal stem cells (MSCs) into the osteogenic lineage [6, 7]. Moreover, the multiple roles of nHA in dentistry include enamel remineralization, dentine hypersensitivity treatment, erosion protection and tooth whitening, while its incorporation into dental implants further enhances material properties for oral rehabilitation [8–10]. Furthermore, nHA has been used as adjuvants in cancer therapy, exhibiting innate anti-tumor properties against various cell lines, including breast, liver and gastric cancers [11, 12]. nHA’s unique nanostructure and chemical composition have shown promising potential in inhibiting tumor growth, primarily through modulating immune system responses [13, 14].

This review discusses the synthesis methods, structure–property relationships and extensive biomedical applications of nHA, delineating its pivotal role in advancing biomaterials research. Various techniques have been developed to modulate the morphology and particle size of nHA, each with its unique advantages and limitations. These methodologies are tailored to optimize nHA’s functionality in tissue regeneration and enhance its anti-tumor efficacy. While synthetic HA nanocrystals share some similarities with natural bone apatite, their differences in formation, size, composition and protein interactions significantly influence their performance in bone tissue regeneration [15]. Furthermore, the exploration of ion-doped nHA is highlighted, which enhances biological and functional properties for applications in bone repair, dentistry and cancer treatment [16]. Specific ions like copper, strontium and lanthanides contribute to antibacterial properties, enhanced biological activity, and unique optical properties [17]. A significant focus is placed on nHA composites, which combine nHA with metals, polymers and bioceramics to address the limitations of pure hydroxyapatite, like brittleness and variable degradation rates. These composites are particularly effective in bone regeneration, where scaffolds integrated with natural polymers closely replicate the structure of native bone [18]. Further research focuses on optimizing scaffold properties to achieve better osteoinductivity, including improving the mechanical behavior and adjusting the degradation rate of the scaffold to match new tissue growth, as well as expanding the use of nanostructured scaffolds to applications ranging from bioimaging and photothermal therapy to tissue engineering and antibacterial infection control [19]. This review provides comprehensive insights into the diverse applications of nHA and its composites in orthopedics, dentistry and tumor treatment, identifies challenges, and encourages discussion for nHA’s future development in the biomedical field.

## Preparation and biomedical application of nHA

### nHA synthesis approaches

nHA is defined as a self-assembled structure of hydroxyapatite, characterized by at least one dimension not exceeding 100 nm in three-dimensional space. This nanomaterial exhibits high surface activity and an ultrafine structure, contributing to its unique properties. nHA can manifest in various forms, including needle-like, rod-like, spherical, and sheet-like shapes, with rod-like and needle-like structures being the most common [20]. Various synthesis methods have been developed to produce nHA, each tailored to yield specific shapes and particle sizes. These methods include the wet chemical precipitation method, biomimetic technique, sol-gel approach, ultrasound and heat treatment, solid phase reaction, spray pyrolysis, hydrothermal treatment and microemulsion techniques. Each method has specific advantages and limitations that profoundly affect the physical properties of nHA and its suitability for specific applications, ranging from tissue engineering to drug delivery systems (Table 1) [38].

**Table 1.** Major processing techniques for producing nHA along with relevant properties

Preparation methods	Shape	Approx. size range (nm)	Cost	Crystallinity	Ca/P ratio	Ref.
Sol-gel approach	Sphere, irregular, rod	30–70, 10–40	Low	Variable	1.83, 1.56, 1.38, 1.20, 1.32	[21, 22]
Wet chemical precipitation	Rod, clusters	64, 100	Low	Frequently low	1.67	[23, 24]
Microemulsion technique	Sphere	<500	High	High	/	[25]
Solid-state reaction	Rod	(15–19) × (26–32)	Low	Very high	1.67	[26]
Hydrothermal treatment	Rod	(40–60) × 20, 10–76	Generally high	Variable	1.67, 1.68	[27, 28]
Template-assisted method	Rod, nano whisker, plate, column	20, 28 × 46, 200–300, 150–250	Low	Variable	1.74, 1.81	[29–31]
Biomimetic technique	Irregular	60–80,	Low	Variable	/	[32]
Heat treatment	Platelet, rod	7 × 18, 50–60, 30–200	Low	Variable	1.2–1.35, 1.74	[33, 34]
Sonochemical method	Flakes, rod	60	Generally low	Variable	1.67	[35]
Ultrasonic irradiation	Irregular, rod, cylindrical, needle, agglomerates	9 × 40, 25, 10 × 30	High	Variable	1.65–1.67	[36, 37]

#### Wet chemical precipitation method 

The wet chemical precipitation method is a widely favored technique for synthesizing nHA due to its simplicity, scalability and cost-effectiveness, which make it ideal for industrial applications. This method involves mixing and precipitating calcium and phosphorus compounds in an alkaline solution [39]. However, it may produce particles with low dispersibility, variable crystallinity and uneven sizes, often in rod, plate or needle-like morphologies. Despite these challenges, this approach is preferred for the bulk production of nHA, especially suitable for standard bone graft applications.

#### Microemulsion technique

The microemulsion technique disperses hydroxyapatite precursors in an oil phase with surfactants, forming micelles that act as nano-reactors to precisely control particle size and morphology [25]. By adjusting the water-to-oil ratio, it manages the hydrolysis of calcium precursors, targeting specific particle shapes. This method produces uniformly sized particles, essential for consistent material properties, but involves complex control of surfactants and their removal. Despite its effectiveness, the higher cost and lower output compared to simpler methods are notable drawbacks.

#### Sol-gel approach

The sol-gel method blends raw materials in a liquid phase, followed by gelation, aging, and heat treatment, producing nanosized powders, fibers or films [40]. This method offers excellent control over the composition and purity of the final product, allowing for the precise incorporation of various dopants. Specific precursors, such as tetraethyl orthosilicate for silica doping, and controlled gelation conditions—temperature, pH and time—significantly influence the properties of the produced nHA. Despite its advantages in particle morphology and size control, the sol-gel process can lead to severe agglomeration.

#### Hydrothermal treatment

The hydrothermal method facilitates nucleation and growth of precursors into specific-sized particles under high temperature and pressure, offering high crystallinity and reduced agglomeration [41]. This technique is favored for producing high-purity nHA, although it is more expensive and produces lower quantities due to the need for complex equipment and higher operational costs. Such factors can limit its feasibility in some production environments. Details such as temperature, pressure, and duration can be specified to highlight the method’s effectiveness in producing high-quality nHA.

#### Template method

The template method employs surfactants introduced beyond their critical micelle concentration into a calcium and phosphate precursor solution [42]. These surfactants self-assemble into specific micelle shapes, serving as templates for controlled nucleation and growth of hydroxyapatite crystals. However, this method faces challenges such as the difficult removal of the templates and potential contamination from toxic by-products. The types of surfactants used, their concentrations and the conditions for template removal, like calcination temperature and atmosphere, are crucial factors that influence the final product’s morphology and purity.

**Figure 1.**
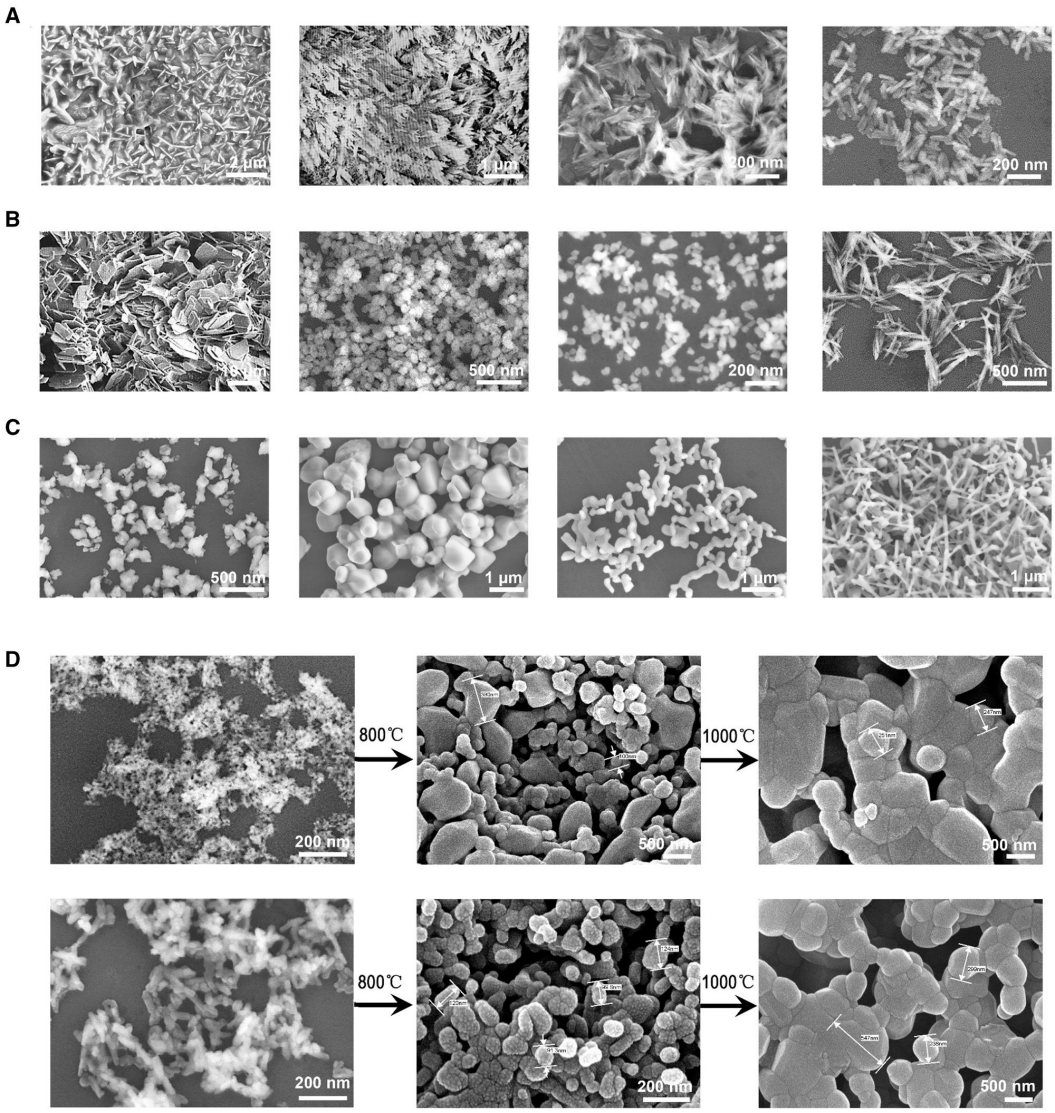
Typical images of nHA crystals. (**A**) nHA particles by wet chemical precipitation. (**B**) nHA particles by hydrothermal treatment. (**C**) nHA particles by heat treatment method. (**D**) nHA particles by precipitation and treated at 800°C and 1000°C for 2 h.

Recent research has focused on combining multiple synthesis methods to achieve enhanced control over the material’s properties, such as morphology and physicochemical characteristics. For instance, needle-shaped nHA particles, measuring approximately 10–15 nm in width and 60–80 nm in length, were obtained from eggshells under alkaline conditions using a wet chemical method assisted by microwave irradiation by Goh *et al.* in 2021 [43]. The extraction from biological sources was an emerging, sustainable approach, aligning with the growing emphasis on eco-friendly manufacturing processes. Another study by Ji *et al.* [44] demonstrated the synthesis of mesoporous hydroxyapatite nanorods using a sodium acetate template via a reflux-hydrothermal method. Similarly, short rod-shaped nHA was prepared using a microwave-hydrothermal method with ultrasonic atomization precipitation [41]. Variations in reaction temperature and time were found to significantly impact crystallinity and diameter, with the concentration of phosphate reactants having negligible effects on crystallinity and dispersion. Our research group has been working on exploring different synthetic method of nHA since 2012. Our findings suggest that wet synthesis methods typically yield nanoparticles with varied shapes like rods, needles and plates, whereas dry methods result in a granular morphology (Figure 1). According to our experience, the choice of phosphate over calcium salts in wet synthesis is crucial in determining nanoparticle shape. Adding surfactants during synthesis not only affects the surface morphology of nHA but also enhances its capacity for drug loading. It is also noteworthy that calcination temperatures exceeding 800°C prompt a transition from nanocrystals to microcrystals, highlighting the critical influence of temperature on the crystalline structure of nHA. For practical applications, an optimal synthesis method should ensure stability, scalability, simplicity, cost-effectiveness and the absence of toxic by-products. The diversity and innovation in nHA synthesis methods profoundly influence its physical form, which in turn significantly impacts its efficacy in a range of biomedical applications.

### Biomedical applications of nHA

For the last decade, numerous studies have emphasized the significant impact of factors like synthesis techniques, particle size, morphology, calcium-phosphorus ratio, concentration and the specific cell line used in experiments on the biological functionality of nHA [45, 46]. It has been observed that smaller particle sizes of nHA can potentially enhance bioactivity and promote a more favorable cellular response [47]. The biomedical applications of nHA, especially in fields such as bone regeneration (e.g. filling bone voids), caries remineralization (e.g. as an active component in toothpaste and dental fillings), and anti-tumor treatments (e.g. as a chemotherapy drug carrier), have been gaining significant attention [8]. One of the primary clinical applications of nHA is in developing bone graft substitutes, where its biocompatibility and osteoconductivity are instrumental in promoting bone growth and healing, especially in cases involving segmental bone defects or large injuries. In dentistry, the use of nHA in implants and coatings is becoming increasingly prevalent, contributing significantly to advancements in implantology and dental prosthetics. However, one of the key challenges with nHA is its mechanical strength, which, while sufficient for non-load-bearing applications, is inadequate for load-bearing orthopedic implants. This necessitates ongoing research into nHA composites reinforced with stronger matrices. Additionally, despite the bioactivity of HA nanocrystals in promoting osteointegration, their recognition as foreign bodies curtails their direct role in bone mineralization [15]. This limitation arises from the inherent differences between synthetic HA nanocrystals and native bone apatite crystals. This highlights the need to develop apatite crystals that more closely resemble native bone apatite, which features variations in crystallinity and surface properties during different stages of bone remodeling.

#### nHA in bone regeneration

Bone is a dynamic, highly vascularized tissue that continuously remodels through bone resorption and formation, a process crucial for repairing significant bone defects caused by trauma, infection or tumor resection [48, 49]. nHA plays a pivotal role in enhancing regenerative performance. Its ability to bind directly to the deficient apatite layer of host bone enhances integration and osteoconductivity, making it an invaluable material in forms such as bone grafts, implant coatings and bone fillers [50]. nHA induces MSCs to differentiate into osteoblasts and accelerates the formation of the calcified cartilage layer and subchondral bone by enhancing the extracellular matrix, primarily type I collagen [51]. Research, including studies by Zhang *et al.* [46], showed that nHA’s effects on MC3T3-E1 pre-osteoblasts were influenced by particle size, dosage, and culture duration. Smaller particles, such as 10 nm nHA, showed a more pronounced effect on cell growth as dosage increases, with the biological impacts regulated by the method of cellular entry. Further studies explored how 20 and 80 nm nHA particles affected human osteoblast MG-63 cells, finding that 20 nm particles significantly enhanced cell proliferation and inhibit apoptosis [52]. Additionally, a fish bone hydrolysis technique was used to produce low crystallinity nHA particles of approximately 19.65 nm, which at concentrations of 50 and 100 μg/ml, improved cell viability and mineralization in MG-63 osteoblasts [53]. Moreover, the surface charge of nHA particles substantially affected their cellular uptake [54]. Studies indicated that positively charged nHA particles promoted superior cell viability and proliferation compared to neutral or unmodified counterparts, emphasizing the importance of surface charge in cellular interactions.

**Figure 2.**
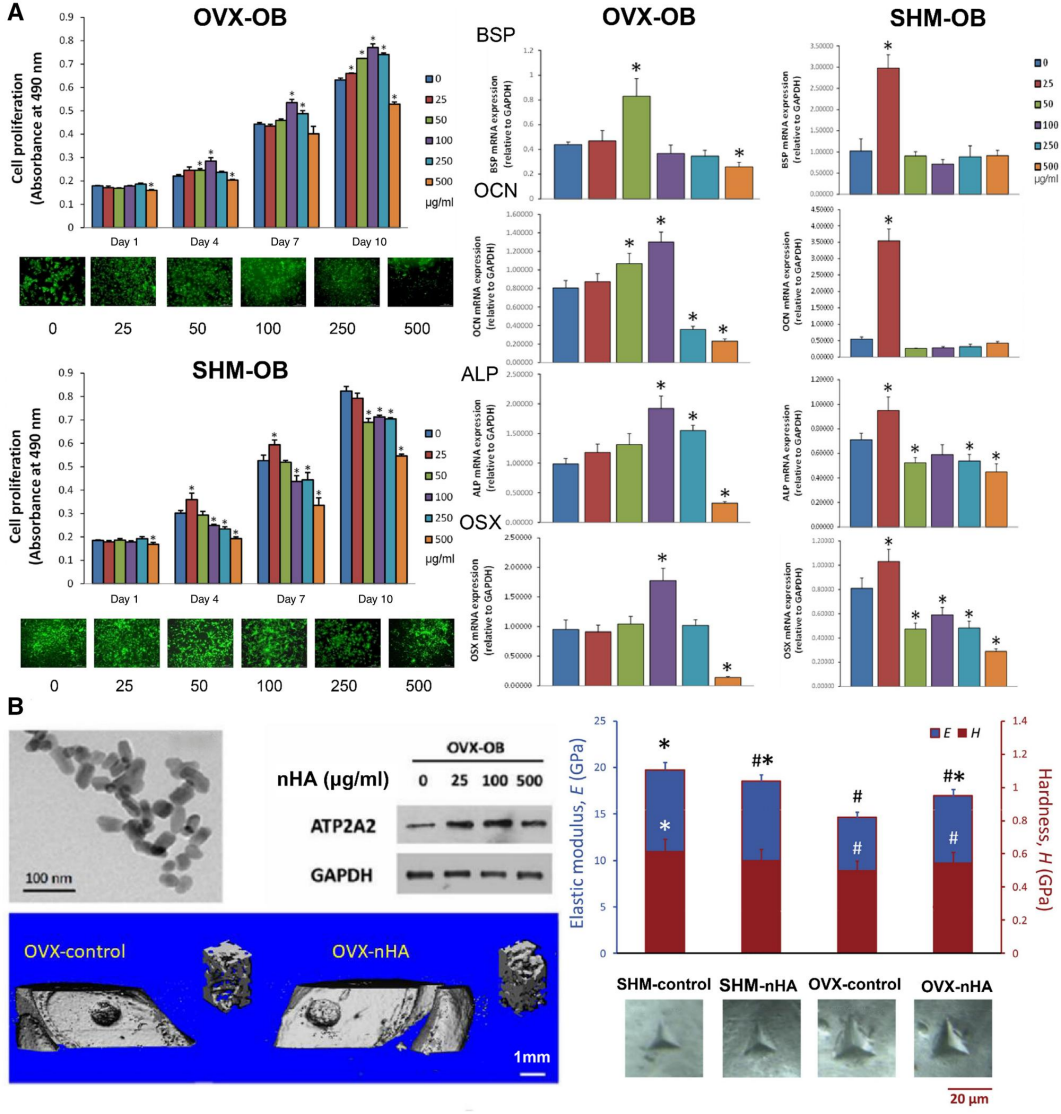
Effect of nHA in osteoporotic bone defects. (**A**) Cell viability of osteoporotic osteoblasts (OVX-OB) and normal osteoblasts (SHM-OB) in different concentrations of nHA as well as bone sialoprotein (BSP), alkali secretion of osteogenesis-related proteins such as sex phosphatase (ALP) and osteocalcin (OCN). #Significant difference from 0 µg/ml nHA treated SHM-OB group (control) with p < 0.05; *Significant difference from 0 µg/ml nHA treated OVX-OB group (control) with p < 0.05. (**B**) *In vivo* evaluation of the distal femurs with drilled defect of OVX and SHM rats, with or without 100 µg/ml nHA treatment after 4 weeks [61]. #Significant difference from SHM-control with p < 0.05; *Significant difference from OVX-control group with p < 0.05. Copyright 2017, Elsevier.

Recent advancements in the preparation of nHA have revealed the significant impact of particle morphology and synthesis methods on its biological functionality. By hydrolyzing a solid precursor crystal of CaHPO_4_ in alkaline solutions with varying pH and ion concentrations, different nHA forms such as nanofibers, nanoneedles and nanosheets were selectively produced [55]. Studies indicated that cellular activities are hindered on surfaces with fine nanoneedles and nanofibers, whereas cells proliferate more effectively on smoother surfaces with larger grains or substrates composed of wider nanosheets. Kalia *et al.* [56] discovered that circular nHA particles were more effective in osteogenic applications compared to rice-shaped nHA, emphasizing the critical role of nHA shape and aspect ratio in new bone formation. Spherical nHA, in particular, demonstrated remarkable potential in bone regeneration [57]. The synthesis technique itself greatly influenced nHA’s properties and efficacy. For instance, nHA synthesized from fish bones using thermal calcination and alkaline hydrolysis methods yielded particles with differing biocompatibility and osteogenic activities [58]. Alkaline hydrolysis hydroxyapatite, with a particle size of 58.3 nm, exhibited higher biocompatibility and osteogenic activity compared to thermal calcination hydroxyapatite (particle size of 64.3 nm). Hydroxyapatite particles of approximately 100 nm were prepared by wet chemical synthesis (WCS) at 37°C and hydrothermal synthesis (HS) at 180°C [59]. Concentrations of WCS and HS particles below 500 μg/ml did not affect cell proliferation or apoptosis but increased the gene expression of alkaline phosphatase (ALP) and bone morphogenetic protein 2 (BMP-2). Zhao *et al.* [60] explored the cellular biocompatibility of two types of nHA, including nanophase standard hydroxyapatite and nanophase calcium-deficient hydroxyapatite (n-CDHA), synthesized by a wet chemical method. The results demonstrated that n-CDHA promoted osteoblast growth and ALP synthesis. Additionally, our research showed that osteoporotic osteoblasts exhibited higher tolerance to nHA concentrations compared to normal osteoblasts, with specific nHA concentrations significantly enhancing osteoporotic bone regeneration *in vivo* (Figure 2) [61].

#### Dental applications of nHA

nHA has emerged as a material of significant interest in dentistry, offering diverse applications in enamel formation, dental adhesives and overall oral health maintenance [62]. Enamel, distinct from other dental tissues, lacks the self-repair ability [63, 64]. Upon exposure to the oral environment, enamel surfaces are rapidly covered by the acquired pellicle, a protective proteinaceous layer formed from salivary components that guards against dental erosion [65]. The introduction of nHA into the oral environment amplifies acid neutralization and remineralization of demineralized lesions [66]. It dissolves within bacterial biofilms, releasing bioavailable ions like calcium and phosphate, which can directly deposit into tooth microporosities, stimulating crystal growth and restoring mineral content [67, 68]. This property highlights nHA’s effectiveness in various dental applications, including dentin remineralization to alleviate tooth sensitivity, caries prevention, biofilm management and tooth whitening enhancements [69–71]. Low concentrations of nHA integrated with whitening agents can effectively achieve remineralization and repair demineralized areas [72]. Toothpaste and mouthwash formulations containing nHA have been developed and shown to be effective in remineralizing artificial dental caries lesions, reducing potential caries depth and forming a new enamel layer [73, 74]. Specifically, nHA precipitated on the enamel surface and remineralized initial enamel caries, resulting in the formation of a new apatite layer with increased enamel surface hardness, thereby preventing tooth decay [75]. Notably, disaggregated nHA regulated oral microecology primarily by inhibiting bacterial growth and metabolism, with minimal impact on the biofilm’s composition [76]. Additionally, incorporating nHA into dental materials has been shown to reduce the wear of resin-modified glass in simulated brushing simulations [77].

The prevention of early root caries is crucial to avoid infection and damage to dental tissues and the alveolar bone, especially considering dentin’s higher susceptibility to acid dissolution compared to enamel [78, 79]. Incorporating nHA into various dental materials, such as root canal sealers, retrofill materials, dental composites and adhesive resin systems, has been shown to enhance their mechanical, physicochemical and biological properties [65]. These advancements have been widely applied in various dental specialties, such as endodontics, periodontics, dental implantology, tissue engineering and restorative dentistry. For instance, the addition of micro- and nano-hydroxyapatite to conventional and resin-modified glass ionomer (RMGI) materials had proven effective in reducing marginal microleakage at the enamel-dentin/bone interface [80]. Incorporating hybrid micro-/nano-hybrid hydroxyapatite rods into reinforced polymer–matrix dental composites led to significant improvements in microhardness, modulus and precipitation ability [81]. In another study conducted in 2022, a resin-based dental restorative composite reinforced with marble dust powder-silane coated nHA exhibited improved mechanical properties, with the composite containing 8% w/w nHA demonstrating maximum hardness, compressive and flexural strength [82]. Furthermore, experimental adhesives incorporating 10% w/w spherical nHA exhibited improved bond strength, facilitating effective dentin interaction and resin tag formation [83]. However, while a 5% w/w nHA concentration in adhesives improved mechanical properties, higher concentrations reduced conversion and shear bond strength [84]. Specifically, the inclusion of 2% w/w nHA in conventional Heliosit/Ivoclar Vivadent adhesive enhanced both the degree of conversion and shear bond strength, whereas 4% w/w nHA reduced these properties in orthodontic adhesive [22]. The size of nHA particles also had emerged as a pivotal factor influencing cell interactions, with smaller particles (∼20 nm) significantly promoting growth effects on odontoblast-like cells over larger particles (∼200 nm) [85]. Titanium implants coated with nHA, produced by selective laser melting, promoted early mineralization, proliferation and osteoblast differentiation of interradicular bone-derived cells [86].

#### nHA in targeted cancer therapeutics

**Figure 3.**
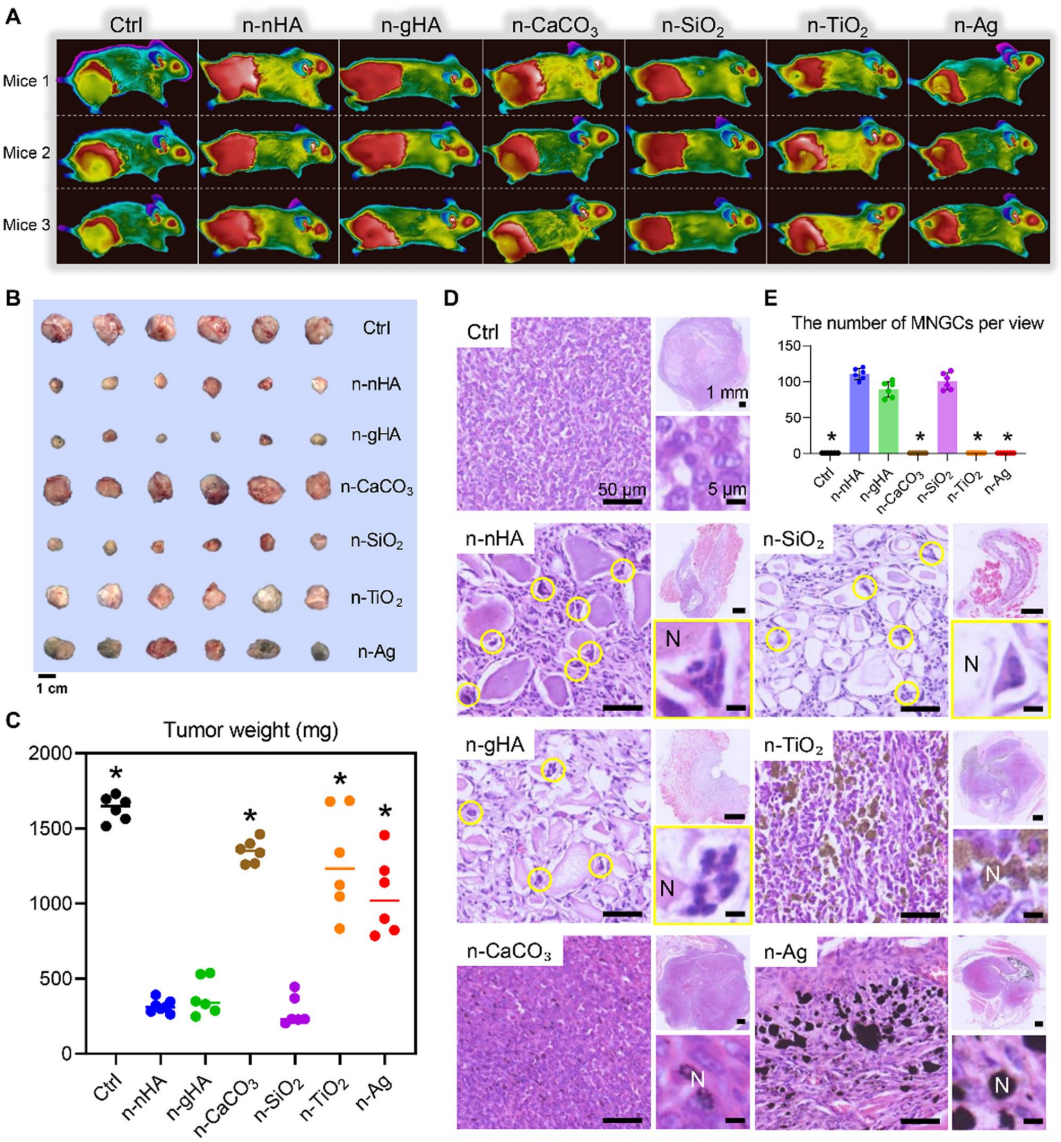
*In vivo* anti-tumor effects of six inorganic nanoparticles. (**A**) Thermal images of tumor-bearing mice after a single injection of different inorganic nanoparticles. Three representative mice from each group are shown at day 28. (**B**) Photographs and (**C**) the measurement of weight of excised tumors at the end of *in vivo* studies. *P < 0.05, significant difference was assessed by one-way analysis of variance (ANOVA) followed by Tukey’s post hoc test compared to n-nHA, n-gHA, and n-SiO_2_ groups. (**D**) Representative hematoxylin and eosin (H&E) staining images of tumor tissues excised at day 28. Top-right images show tumor tissues at low magnification. Left images show the appearance of nanoparticle aggregates in tumors. Bottom-right images show the highly magnified cells surrounding the nanoparticle aggregates. N, aggregates of nanoparticles. Circles indicate the multinucleated giant cells (MNGCs). (**E**) Average number of MNGCs counted from 100-fold histology images of tumors representative of three independent mice [13]. *P < 0.05, significant difference was assessed by one-way ANOVA followed by Tukey’s post hoc test compared to n-nHA, n-gHA, and n-SiO_2_ groups. Ctrl, control. Copyright 2023, Science Advances.

Cancer treatment has undergone significant evolution in recent years, shifting towards targeted therapies that aim to minimize the severe toxic effects on healthy tissues traditionally associated with surgery, chemotherapy and radiotherapy [87]. Emerging therapeutic strategies, such as gene therapy, immunotherapy and photothermal therapy, have opened new avenues for cancer treatment [88, 89]. When combined with nanomaterials, these therapies have shown enhanced efficacy and complementary benefits. nHA has gained attention in the oncological field due to its precise targeting abilities. It has shown promising results in selectively inhibiting proliferation and inducing apoptosis in various cancer cell types such as osteosarcoma, breast, gastric, colon and liver cancer, while sparing healthy cells [90]. A study by Tang *et al.* [91] highlighted nHA’s selective toxicity towards gastric, cervical adenocarcinoma and liver cancer cells, exploiting the electrolyte and metabolic vulnerabilities of tumor cells. The physicochemical properties of nHA, such as morphology, particle size, concentration and crystallinity, play a critical role in regulating tumor growth [92]. For example, Fukada *et al.* [12] found that nHA with a particle size less than 50 nm exhibited high inhibitory activity on breast cancer cells while having minimal impact on normal cells. Sun *et al.* [93] reported that rod-shaped nHA, approximately 10 nm in width and 50 nm in length, suppressed lung cancer cell activity through mitochondrial-mediated apoptosis in a dose- and time-dependent manner. Wang *et al.* [94] in 2022 observed that different concentrations of nHA did not affect the adhesion of osteosarcoma cells but significantly inhibited their proliferation. Furthermore, Wu *et al.* [95] explored the effects of nHA on melanoma cells, identifying the granular shape, small size, high specific surface area and low crystallinity as key factors in its potent anti-tumor activity. Research into the anti-tumor mechanisms of nHA has advanced significantly, revealing intricate pathways and cellular processes. The anti-tumor effects of nHA are underpinned by three key mechanisms:

1. Enhanced permeability and foreign body response: nHA aggregates at tumor sites due to retention (EPR) effects and enters tumor cells via receptor-mediated endocytosis [96, 97]. One study conducted by our research group in 2019 by Zhang *et al.* [90] suggested that rod-shaped nHA selectively inhibited bone tumor cell proliferation through specific particle entry behaviors, initiating both endogenous and exogenous apoptosis pathways. In 2023, our further research demonstrated the inhibition of tumor growth, prevention of metastasis and increased survival rates in tumor-bearing rabbits treated with hydroxyapatite nanorods. Needle-shaped nHA, granular nHA and silica have also been proven effective in inhibiting tumor growth *in vivo* [13]. We found nHA modulates STXBP6 protein expression, induces autophagy, and facilitates macrophage fusion into multinucleated giant cells, thereby inhibiting tumor growth and metastasis (Figure 3).
2. Interaction with the endoplasmic reticulum: nHA, taken up by tumor cells via endocytosis, is mainly localized in the endoplasmic reticulum, responsible for protein and lipid synthesis in tumor cells [98]. nHA interacts with ribosomes attached to the endoplasmic reticulum to reduce the binding of messenger ribonucleic acid (mRNA) to the ribosomes [92]. This interaction inhibits protein synthesis and blocks the cell cycle in the G0/G1 phase, thereby promoting apoptosis. For example, chemically assisted ultrasound irradiation was used to produce rod-like nHA with a size of 67.6 nm, which induced cell aggregation in the G1 phase, blocked cell cycle progression and inhibited the proliferation of liver cancer cells (BEL-7402) *in vitro* [99]. Guo *et al.* [100] demonstrated that nHA induced dose-dependent apoptosis and G2/M arrest in rat glioma C6 cells and human glioma U87MG ATCC cells, potentially through the downregulation of NF-KB signaling.
3. Apoptosis mediated by mitochondria: nHA releases calcium ions, disrupting the intracellular calcium balance and triggering a cellular oxidative stress response [92, 101–103]. This disturbation activates both mitochondrial-mediated cell apoptosis and caspase apoptosis pathways. Previous studies have shown that spherical nHA in the size range of 10–30 nm induces apoptosis of tumor cells by activating the mitochondrial-dependent apoptosis pathway and negatively regulating the phosphatidylinositol-3-kinase/protein kinase B (PIK3/AKT) pathway [104]. Various forms of nHA, including granular, rod-shaped, and needle-shaped, were internalized into melanoma cells [95, 105]. Granular nHA, in particular, induced changes in mitochondrial morphology and a loss of mitochondrial membrane potential, demonstrating high intracellular efficiency in tumor cells. In tumor-bearing mouse models, all forms of nHA delayed tumor growth, with granular nHA showing the highest efficacy.

The potential of nHA in cancer therapy extends beyond treatment to early detection, reducing therapy toxicity, and overcoming resistance to existing treatments [92]. Innovative applications of nHA as imaging probes and drug carriers are promising avenues for advancing cancer therapeutics. For example, Zhang *et al.* [106] developed polyacrylic acid-coordinated nHA (HAP-PAA) and further functionalized them with folic acid for cancer therapy. Their study showed that spherical HAP-PAA nanoparticles with low crystallinity exhibited substantial lethality against tumor cells. After functionalizing the nanoparticles with folic acid for targeted delivery, their cellular uptake and specific cytotoxicity against tumor cells were significantly enhanced. In addition, a novel vaccine was developed that induced an anti-EGF immune response through the covalent interaction between nHA (average size of 60 ± 10 nm) and proteins [107]. This immune response persisted throughout the 91-day treatment period after implantation in mice, offering the potential for long-term treatment of cancer originating from epithelial tissues.

### Ion-doped nanohydroxyapatite in biomedical applications

To more closely mimic the composition of natural bone minerals, researchers have been incorporating a variety of elements from the periodic table into hydroxyapatite’s structure using primarily wet chemical methods [108]. These elements include a variety of ions like strontium (Sr^2+^), magnesium (Mg^2+^), copper (Cu^2+^) and lanthanum (La^2+^), which can substitute for calcium ions (Ca^2+^) within the crystal lattice of hydroxyapatite [109]. Similarly, anions such as carbonate (${\text{CO}}_{3}^{\;2-}$), selenate (${\text{SeO}}_{4}^{\;2-}$), silicate (${\text{SiO}}_{4}^{\;2-}$), fluoride (F^−^) and chloride (Cl^−^) are substituted for phosphate (${\text{PO}}_{4}^{\;3-}$) and/or hydroxide (OH^-^) ions, introducing unique properties tailored to specific biological functions [110]. Additionally, doping with antibacterial ions such as silver (Ag^+^), Cu^2+^, and zinc (Zn^2+^) has been explored to impart hydroxyapatite with antibacterial properties [111]. The introduction of ions such as Mg^2+^, Sr^2+^ and Zn^2+^ has been shown to enhance the biological activity and physicochemical properties [112]. Notably, the incorporation of lanthanide element ions such as terbium (Tb^3+^), erbium (Er^3+^), europium (Eu^3+^), La^3+^ or dysprosium (Dy^3+^) into hydroxyapatite enhances its optical properties, making it suitable for applications in luminescent materials, biological imaging, biological response probes and sensors (Table 2) [124, 125].

**Table 2.** Ion-doped nHA and the enhanced biological function studies

Ion	Method	Morphology	Particle size (nm)	Ion ratio	Cell/animal	Function	Refs
Sr^2+^	Hydrothermal	Rod	<100	/	MG63	Excellent biological performance both in cell proliferation and differentiation	[113]
Mg^2+^	Wet precipitation	Plate	50–80	2.2 mol%	MC3T3-E1	Enhanced spreading and proliferation of osteoblasts	[114]
Zn^2+^	Microwave assisted	Rod	40–52	1, 2.5, 5 mol%	MDA MB 231 cells	Low-rate Zn-doped HA enhanced the radiation effect on breast cancer cells	[115]
	Mechanochemical process	Flaky	82	/	/	Increased antibacterial activity	[116]
Rb^+^	Hydrothermal treatment	Rod	50–300	1, 3, 5, 7, 10 mol%	MG-63	3%Rb-nHAp promoted proliferation and ALP activity in MG-63 cells. Rb-nHAp powders showed strong antibacterial activity	[117]
Zr^4+^	/	Rod	30–50	/	A549	Exhibited a cytotoxic profile in lung cancer cells	[118]
F^-^	Precipitation	Irregular	200–400	1, 2, 3%	L929 fibroblast cells	All nHA showed effective tubule occlusion. The addition of fluoride enhanced the chemical stability and solubility properties, with optimal results observed at a fluoride concentration up to 2%	[119]
Co^2+^	Wet chemical precipitation	Rod	31.23 ± 6.04, 26.62 ± 8.88, 23.82 ± 5.1, 21.99 ± 6.34	0.5, 1, 2 mol%	MDA-MB-231 cell	2.0 mol% Co-HAp showed optimal biological and fluorescence performance. Cobalt doping enhanced the fluorescence intensity of FITC-labeled HAp and facilitated improved DOX loading and sustainable pH-regulated release	[120]
Fe^2+^	Microwave-assisted synthesis	Particles, thread-like, spherical core-shell	(5–10) × (200–300), (5–10) × (300–400), 50–100	0.5, 2.6, 6.4 mol%	MG63	Fe-doped HAp demonstrated a non-toxic nature on MG63 cells and showed excellent antibacterial susceptibility against selected pathogens	[121]
Cu^2+^	Wet chemical precipitation	Short rod, sphere	20 − 40	4, 8, 16, 20, 25 mol%	Endothelial and osteoblast cells, rat cranium	A positive correlation was found between copper content and the inhibition of bacterial growth. Endothelial and osteoblast cells proliferated rapidly on γ-PGA/CuxHAp. 20 mol % Cu ions reduced cell proliferation. Certain γ-PGA/CuxHAp samples exhibited efficient bone regeneration capacities at 12 weeks post-implantation	[122]
Ce^3+^	Microwave-assisted hydrothermal treatment	Rod	(10–80) × (50–450)	1, 3, 5%	/	Inhibited bacteria on *S. aureus*, *E. faecalis*, and *C. albicans strains*	[108]
Eu^3+^	Sol-gel assisted precipitation	Sphere	50–100	3, 6, 10 mol%	Kidney cell lines HEK-293	Enable bioimaging, good antimicrobial activity and cell viability	[123]

#### Ion-doped nHA for bone repair

Hydroxyapatite crystals enriched with calcium and phosphate, are complemented by trace elements such as magnesium, silicon, strontium and fluorine, playing a vital role in maintaining bone remodling [126]. These trace elements are essential for the growth and development of human bone tissue; insufficient levels can significantly increase the risk of osteoporosis and fractures. Considering this, researchers have incorporated various trace elements into nHA to significantly enhance its osteogenic performance [127]. For instance, boron-containing nHA composites showed enhanced osteogenic differentiation of MSCs, indicated by increased ALP activity, surpassing results with nHA composite and boric acid alone [128]. Transcriptomic analyses reveal that boron influences genes in the Wnt and TGF-β signaling pathways and enhances cellular stress responses. HS of strontium-substituted hydroxyapatite (SrHA) has revealed that SrHA nanorods, especially those over 100 nm in length, significantly promote cell proliferation and osteogenic differentiation [113]. Gene expressions related to osteogenesis, such as ALP, OCN and Runt-related transcription factor 2, were consistently higher in SrHA than in HA, highlighting its potential for improved bone regeneration. Further research by Maleki *et al.* [116] involved preparing zinc-doped nHA with a flake structure using a solid-state reaction method, which notably enhanced MSCs proliferation and differentiation. Additionally, short rod-shaped nHA doped with varying amounts of rubidium (Rb-nHA) synthesized via hydrothermal methods showed improved proliferation and differentiation of MG-63 cells and exhibited superior antibacterial properties compared to pure hydroxyapatite [117]. Shu *et al.* [122] developed a composite material, γ-polyglutamic acid, and copper co-synthesized hydroxyapatite (γ-PGA/CuxHAp). This material demonstrated a positive correlation between copper content and bacterial growth inhibition, with smaller nanoparticles exhibiting higher antibacterial activity. At low concentrations, copper-substituted γ-PGA/CuxHAp also promoted the proliferation of endothelial cells and osteoblasts. In a rat calvarial defect model, specific γ-PGA/CuxHAp samples significantly enhanced bone regeneration 12 weeks after implantation.

The synthesis of multi-ion doped nHA materials has become the focus of extensive research, aiming to replicate the complex chemical composition and structural properties of natural apatite born in physiological environment [129, 130]. These efforts involve diverse and innovative synthesis approaches, each tailored to enhance osteogenic differentiation and bone regeneration capabilities. One such approach involved a biomimetic self-assembly method mediated by collagen templates to synthesize rod-shaped Zn/Sr dual ion-collagen co-assembled hydroxyapatite (ZnSr-Col-HA) [131]. This scaffold demonstrated a profound impact on the osteogenic differentiation of MSCs *in vitro*, emphasizing the critical role of the osteo-immune microenvironment and the dynamic effects of ion composition on osteogenic gene expression. In another study, macroporous chitosan-agarose bone scaffolds comprising Mg- and Zn-doped nHA (chit/aga/HA) were developed as scaffold materials [114]. The incorporation of Mg^2+^ enhanced osteoblast spreading, cell proliferation and osteocalcin production, indicating its positive influence on early osteogenic markers. In contrast, Zn^2+^ improved type I collagen production and extracellular matrix mineralization but did not significantly enhance cell proliferation or osteogenic differentiation, as evidenced by the decreased levels of ALP and osteocalcin in cells exposed to Zn^2+^. Furthermore, a study using a microwave reflux process to incorporate ferric (Fe^3+^) and selenate (${\text{SeO}}_{4}^{\;2-}$) ions into the hydroxyapatite structure (Fe-SeHA) produced granular nanoparticles that enhanced the adhesion and proliferation of human fetal osteoblasts (hFOB) and exhibited higher ALP activity [132].

#### Ion-doped nHA in dental application

In the fields of dental and orthopedic repair, the requirements for inorganic materials significantly differ due to the distinct physiological environments and functional demands of each application. One of the significant advantages of nHA lies in its versatility, as various ions can be incorporated during the synthesis process, profoundly influencing its properties. Ion doping intricately influences the crystal nucleation, growth, orientation and solubility, thereby mimicking the behavior of biological apatite [133–135]. The controlled release of ions from nHA has been demonstrated to effectively reduce caries lesions on marginally sealed teeth, showing its potential in preventive dentistry [136]. For instance, nHA doped with 25 and 50 mol% Sr inhibited incipient carious lesions effectively [137]. Tooth samples treated with Sr-doped nHA exhibited enhanced surface roughness and improved performance in microindentation tests. Formulations containing 1%, 2%, and 3% F-doped nHA have effectively blocked dentin tubules, achieving complete occlusion of dentinal tubules with significantly greater penetration depth [119]. Furthermore, Mg-doped nHA, synthesized through precipitation methods, was found in 2023 to improve interactions between dentin and adhesive materials, enhancing bond strength and the integrity of the resin-dentin bond while also imparting antibiofilm properties [138]. Zn-doped hydroxyapatite, synthesized into round particulates via chemical co-precipitation, demonstrated enhanced biological and antibacterial activities [139]. An increase in Zn^2+^ concentration reduced the crystal size of hydroxyapatite, thereby amplifying Zn’s antibacterial effectiveness against bacteria such as *Staphylococcus aureus* (*S. aureus*) and *Escherichia* sp. Additionally, the substitution of ${\text{CO}}_{3}^{2-}$ for ${\text{PO}}_{4}^{3-}$ within the hydroxyapatite lattice led to alterations in crystal structure, solubility and mechanical properties [140, 141]. This substitution resulted in a decrease in enamel crystallinity alongside an increase in carbonate content. Researchers synthesized non-stoichiometric nanocrystalline hydroxyapatite with minimal carbonate content, demonstrating its potential as dental material [142]. Recent clinical trials comparing nanostructured carbonate hydroxyapatite (CHA) with commercially available bovine xenograft have revealed CHA’s higher biodegradation rate [143]. This characteristic made it a highly promising biomaterial for alveolar socket preservation before implant treatment.

Biological tooth apatites, characterized as carbonated non-stoichiometric and Ca-deficient compounds, often contain trace elements, either adsorbed on the crystal surface or within the lattice structure [144]. Replicating the multifaceted biological functions of apatites through single-atom doping presents significant challenges due to the complexity of their natural composition [142]. However, strategic multi-ion substitution within the apatite structure can create synergistic effects, enhancing the bioactivity and functionality of synthetic apatites. For instance, a study by Hassan *et al.* [145] demonstrated a rod-shaped Sr/Zn co-doped nHA and poly(lactic-co-glycolic acid) (PLGA) composite scaffold that exhibited significant antibacterial activity against *Staphylococcus aureus* and supported osteoblast proliferation. Another example includes cerium (Ce^3+^) and Mg^2+^ co-doped hydroxyapatite nanorods synthesized via microwave-assisted hydrothermal treatment, which effectively inhibited bacteria, including *S. aureus*, *E. faecalis*, and *C. albicans* strains [108]. While ion doping enhances the biofunctional properties of apatite, excessive ion doping can alter the physical properties of apatite, potentially resulting in adverse effects. For instance, the formation of ${\text{CO}}_{3}^{2-}$ substituted hydroxyapatite with high ${\text{CO}}_{3}^{2-}$ content increased the solubility of tooth enamel, leading to dental caries [142]. Excessive fluoride intake during tooth development could lead to dental fluorosis [146]. Therefore, maintaining a balanced approach is essential when considering ion doping in nHA-based materials to ensure both efficacy and safety in various applications.

#### Ion-doped nHA in cancer therapy

Various modification techniques have been used to transform nHA into smart anticancer agents capable of selectively targeting and eradicating cancer cells while minimizing adverse effects on healthy tissues. Ion doping has become a critical strategy in developing these multifunctional biomedical materials [115, 121]. For example, Febrian *et al.* [118] in 2021 synthesized 30–50 nm rod-shaped zirconium-doped hydroxyapatite (HAp-Zr) nanoparticles for lung cancer therapy. The study demonstrated that HAp-Zr accumulated in lung cancer cells (A549 cell line) due to strategic zirconium doping, resulting in enhanced cellular internalization. Barbarente *et al.* [147] prepared plate-shaped nanoparticles of selenium-doped hydroxyapatite (HASe) with Se/(P + Se) molar ratios (ranging from 0.01 to 0.40) using a mild wet method. Their findings revealed that higher selenium doping levels in HASe exerted cytotoxic effects on both cancerous and healthy cells. Additionally, the development of superparamagnetic nHA through the incorporation of magnetic ions such as Fe^2+^ and gadolinium (Gd^3+^) offers potential applications in cancer treatment and diagnosis [148–150]. Doan *et al.* [120] in 2022 prepared short rod-shaped nanoparticles of cobalt-doped hydroxyapatite (Co-HAp) using a wet chemical precipitation method. The 2.0 mol% Co-HAp nanoparticles exhibited excellent fluorescence sensitivity when conjugated with FITC and applied to MDA-MB-231 cell lines without toxicity. Additionally, Zeng *et al.* [151] developed a dual-modal (CT/fluorescence) bioimaging probe based on barium-doped nHA with near-infrared (NIR) emission around 700 nm. *In vitro* CT and fluorescence cell imaging of the conjugates demonstrated dual-modal bioimaging and tumor targeting capabilities.

Cancer research innovations have led to the development of multi-ion doped nHA, designed to augment therapeutic efficacy and incorporate it into advanced cancer diagnosis and treatment modalities [152, 153]. For example, rod-shaped Mn/F double-doped mesoporous nHA with paramagnetic properties was synthesized for nuclear magnetic resonance imaging [154]. The pH-responsive release, combined with the enhancement of Mn^2+^ and the relaxation of protein complexation, enhanced the inhibitory effect of chemotherapy drugs on HeLa cells and improved the signal contrast between the tumor microenvironment and normal tissues. Mansour *et al.* [155] synthesized palladium (II) and sulfate-substituted hydroxyapatite nanoparticles (∼30 nm) using a microwave-assisted co-precipitation method, which increased cell mortality in lung cancer (A549) cells and effectively induced their degeneration. Recent interest has surged in nHA-based dual-/multi-modal bioimaging systems, particularly those incorporating ions like Eu^3+^, Gd^3+^ and Mn^2+^ for enhanced imaging properties. Eu/Gd co-doped hydroxyapatite (HAP: Eu/Gd) nanocrystals, synthesized by coprecipitation while maintaining a plate-like shape, demonstrated successful cell labeling and *in vivo* imaging capabilities, remaining detectable in the bloodstream for at least 3 h post-injection [156]. Binary nHA-based CT contrast agents incorporating barium (Ba^2+^) and holmium (Ho^3+^) have shown improved CT contrast efficiency compared to undoped nHA [157]. In another study, Eu/Ba co-doped and F-substituted nHA (HA@nFAp: Eu/Ba) with spindle-like morphology was prepared, exhibiting high sensitivity in both CT and fluorescence imaging and stable tumor-targeting properties in cancer cells and living mice [158].

## nHA composites: preparation and biomedical applications

### Component of nHA composites

While nHA is inherently bioactive, its standalone pristine form as slurry or powder often exhibits insufficient mechanical strength and slow degradation rates, limiting its practical application in biomedicine. To improve their mechanical and bioactive properties, nHA is often integrated with metals, polymers and bioceramics, enhancing attributes like strength, flexibility and thermal stability [159]. Addressing these requires advanced biocompatibility screening, consistent manufacturing techniques such as 3D printing and proactive regulatory engagement. Strategic solutions include refining the microarchitecture of composites to enhance integration with host tissue and collaborating with regulatory bodies to establish specific standards for biomaterial composites. These efforts are crucial for tailoring nHA composites to clinical needs and facilitating their successful adoption in medical applications.

#### Metal scaffolds with nHA

Metal materials, such as titanium (Ti) and its alloys, tantalum and magnesium and its alloys, are extensively used in biomedical implants [160–162]. In biomedical implants, nHA coating plays a crucial role in enhancing the bioactivity of metal implants, thereby improving their functionality and applicability in clinical applicability [163]. A diverse array of techniques is employed for coating metal surfaces with nHA, ranging from wet processes like electrochemical deposition and sol-gel to dry processes including sputtering and plasma spraying [164]. Notably, plasma spraying stands out as the sole FDA-approved method for biomedical coatings, offering remarkable properties like dense uniformity, strong bonding and high efficiency [165]. However, challenges persist, particularly in optimizing the mechanical properties and adhesion of nHA coatings to metal substrates. To address these challenges, researchers have developed innovative techniques, such as modifying porous Ti surfaces by vacuum spraying to improve adhesion, sustained release and mineralization [166]. Another study in 2021 used a modified electrophoretic deposition to form a bone-like hydroxyapatite/collagen nanocomposite (HAp/Col) on a Ti substrate with controllable thickness and high adhesion strength on the Ti surface [167]. MG63 cells cultured on this composite showed comparable proliferation rates and superior ALP activity compared to those on bare Ti. Additionally, a nanorod-structured hydroxyapatite coating with a pure phase and high crystallinity was achieved by combining atmospheric plasma spraying with hydrothermal treatment [168]. This nano surface enhanced the attachment, proliferation and differentiation of MSCs, significantly improving the osseointegration between implants and surrounding bone tissue.

**Figure 4.**
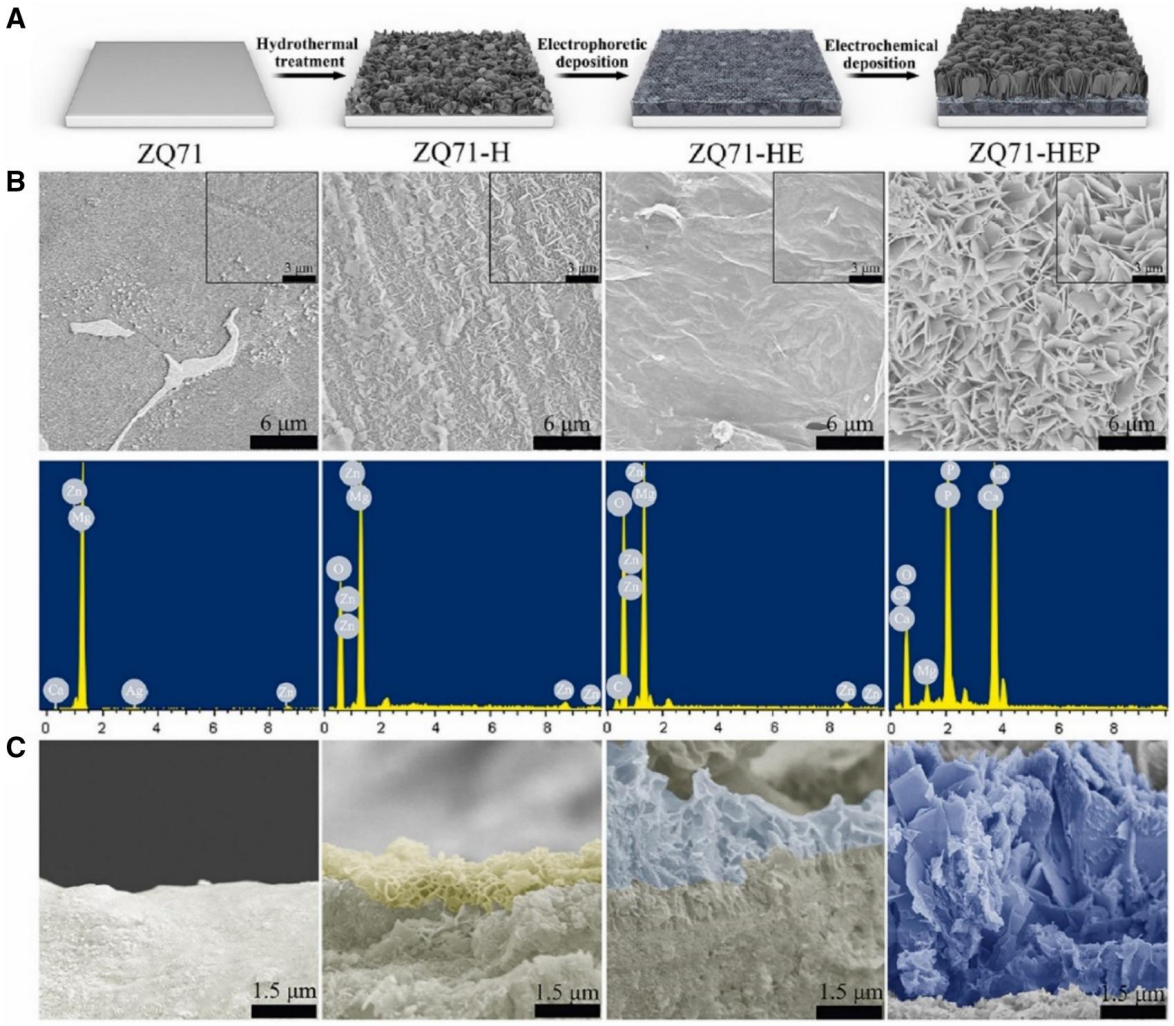
Preparation and morphological characterization of various ZQ71 samples. (**A**) Schematic illustration of the preparation of various ZQ71 samples. (**B**) Surface SEM images and EDS analysis and (**C**) cross-section SEM images of various ZQ71 samples [178]. Copyright 2023, Elsevier.

Magnesium, zinc and their alloys recently have gained attention as promising alternatives in implant technology due to their excellent biocompatibility, mechanical compatibility and degradability [169, 170]. However, the rapid degradation of magnesium, especially through localized corrosion under physiological conditions, presents a significant challenge that limits its clinical adoption in areas such as orthopedic implants [171]. Researchers have focused on enhancing the corrosion resistance of these alloys through various surface treatment technologies, including electrochemical deposition, anodization and microarc oxidation [172, 173]. Hydroxyapatite coatings have been shown to slow down localized degradation, making magnesium and zinc alloys more suitable for load-bearing orthopedic implants [174–176]. For example, a biomimetic fluoridated hydroxyapatite (FHA) coating, composed of bilayer arrays of nanoneedles with micro-/nano-topography, was formed on magnesium alloy surfaces using a microwave aqueous method [177]. *In vitro* biological tests demonstrated that this FHA coating enhanced the osteogenic differentiation capacity compared to conventional hydroxyapatite coatings, providing long-term protection for magnesium alloy. Another approach involved applying a nanocomposite coating, composed of inner magnesium hydroxide, middle graphene oxide and outer hydroxyapatite (Mg(OH)_2_/GO/HA) on the surface of magnesium alloys (ZQ71) (Figure 4) [178]. This composite was achieved through a combined approach of hydrothermal treatment, electrophoretic deposition and electrochemical deposition. All three coatings exhibited high bonding strength, hydrophilicity and corrosion resistance, with the outer hydroxyapatite layer displaying excellent osteogenic activity. *In vivo* experiments confirmed that this composite nHA coating regulates the degradation rate of magnesium and its alloys while enhancing their biological activity.

#### Polymer-nHA composite scaffolds

**Figure 5.**
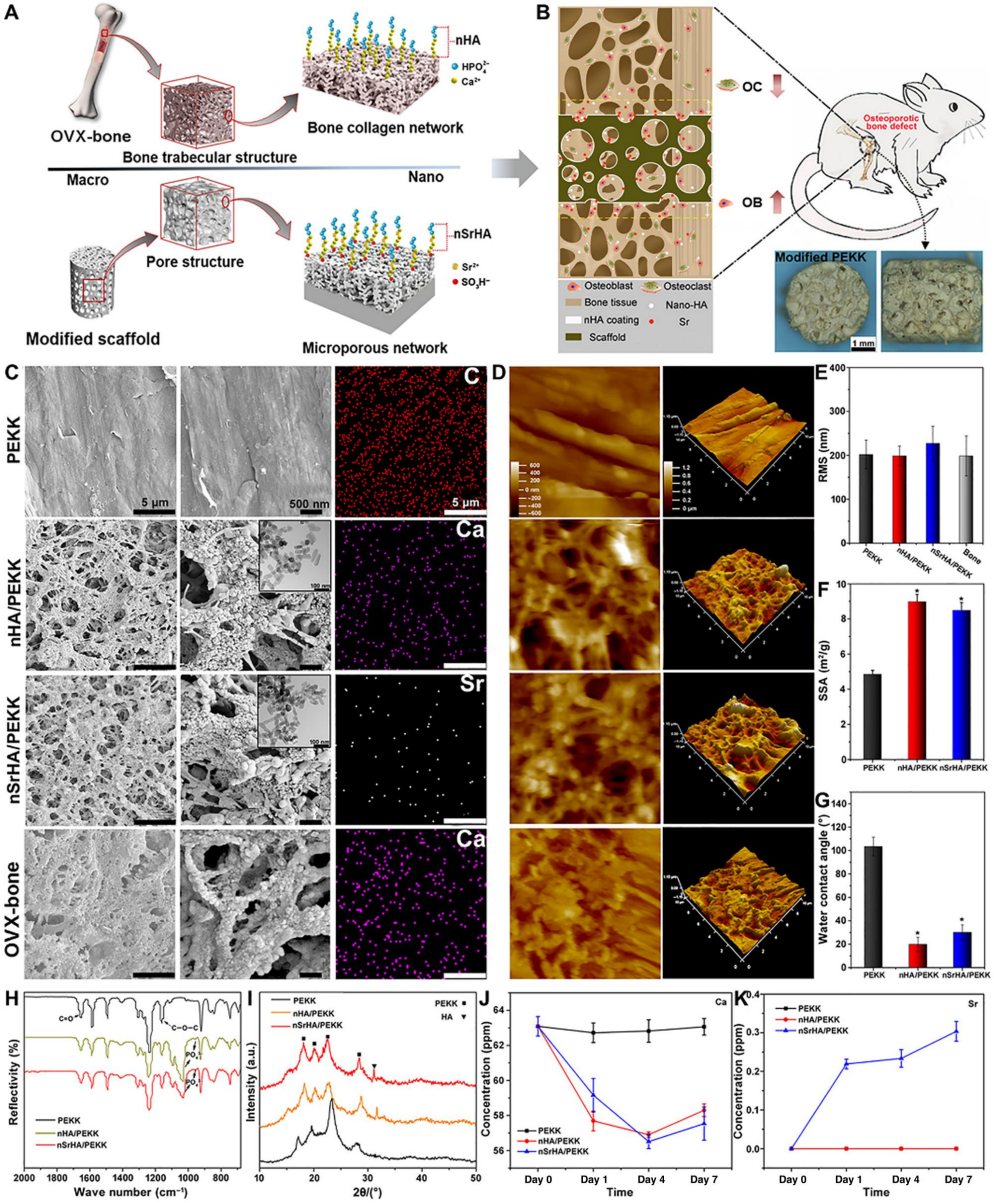
Preparation and characterization of the biomimetic PEKK materials. (**A**) Schematic illustration of the preparation of biomimetic PEKK scaffold. (**B**) Regulating effect of the biomimetic PEKK scaffold on osteogenesis and osteoclastogenesis of osteoporotic bone defect. OC, osteoclast; OB, osteoblast. (**C**) Typical scanning electron microscopy (SEM) and transmission electron microscopy (TEM) images and element mapping of various PEKK scaffolds and natural osteoporotic bone. (**D**) Atomic force microscopy (AFM) images and corresponding (**E**) surface roughness of PEKK scaffolds and natural osteoporotic bone. (**F**) Specific surface area (SSA) of different scaffolds. (**G**) Water contact angle, (**H**) Fourier transform infrared spectrophotometer (FTIR) spectra, and (**I**) X-ray diffractometer (XRD) patterns of different scaffolds. (**J**) Ca and (**K**) Sr ion release behavior of different scaffolds in cell culture medium [184]. Copyright 2020, Science Advances.

Medical polymer materials, integral to various biomedical applications, span a wide range from non-degradable materials such as polyethylene (PE), polypropylene and polyacrylate, to degradable biomaterials, including natural polymers like collagen and cellulose, and synthetic polymers such as polyvinyl alcohol, polylactic acid (PLA) and PCL [179, 180]. These materials are selected based on their unique properties, tailored to specific medical applications. For medical implants, polymers with superior mechanical properties and ease of processing are preferred, such as high-density PE, polyetheretherketone (PEEK) and polyamide [181]. Ensuring strong interfacial adhesion between bioinert polymer materials and host bone is paramount to avert fibrous tissue encapsulation, a phenomenon that could culminate in implant loosening and subsequent failure [182]. Various techniques, including physical coating, mechanical mixing, melt mixing and *in situ* controlled radical polymerization, have been used to prepare polymer/inorganic nanocomposites to enhance the functionality of polymer materials [183]. For example, a rod-like strontium-doped nHA coating was applied to the surface of a polyether ketone ketone (PEKK) scaffold using the wet chemical deposition method (Figure 5) [184]. *In vitro* and *in vivo* studies demonstrated that this scaffold promoted osteoporotic bone regeneration and delayed adjacent bone loss by regulating osteoblasts and osteoclasts. Another bioactive coating of nHA on PEEK prepared using the sonocoating method conducted in 2021 was shown to enhance the drug absorption of cefuroxime sodium salt while exhibiting potent antibacterial properties against *S. aureus* [185]. Additionally, a two-part injectable system consisting of nHA, silver salt (Ag_3_PO_4_) and polyurethane with varying nHA and Ag_3_PO_4_ contents was developed [186]. Increasing the nHA content from 0 to 30 wt% enhanced compressive strength, setting time, surface wettability and differentiation activity.

The degradation of biodegradable polymer materials such as PLA and PCL can lead to a decline in the interface pH value, potentially influencing the desired biological interactions essential like osteoblast differentiation [187]. The stabilization of microenvironment’s pH occurs as nHA’s degradation products neutralize the acidic byproducts of PLA or PLGA, thereby facilitating a more conducive environment for cell growth and differentiation [188]. Embedding 10–30% nHA in PLA microspheres using the emulsion solvent evaporation method, led to significant improvements in these materials [189]. These enhancements included increased protein adsorption capacity and proliferation, attributable to the added nHA, which also imparted increased surface hydrophilicity and roughness. Nano-hybrid composites also demonstrated superior mechanical properties, including enhanced compressive and tensile strength, as well as reduced polymerization shrinkage [190–192]. For example, biodegradable polymers (PDLLA/PLGA) coated with nHA using sonocoating and electrospraying methods showed that the ultrasonic nHA coating enhances structural stability, mitigates harmful pH changes and improves wetting properties while extending membrane degradation time [193]. These sonocoated membranes exhibited mechanical properties comparable to those of other commercially available membranes. To address the issue of poor interfacial bonding between polymer and hydroxyapatite, one attempt made in 2022 involves grafting poly(D-lactide) (PDLA) onto the surface of nHA to form grafted hydroxyapatite (g-HAP) [194]. Subsequently incorporating g-HAP into PLLA significantly enhanced interfacial bonding. This approach, employed in scaffold fabrication using selective laser sintering (SLS), demonstrated improved mechanical properties and biocompatibility.

Natural polymers, including polysaccharides (such as chitosan, cellulose, hyaluronic acid, alginate, etc.), proteins (such as collagen, fibrin, etc.) and heterologous derived materials, are widely used due to their excellent biocompatibility [195]. However, their inherent instability and proneness to degradation often limit clinical utility. Incorporating nHA into natural polymer scaffolds offers a strategic solution to these challenges by enhancing mechanical strength, reducing degradation rates and improving osteogenic performance, thereby closely mimicking the composition and structure of natural bone minerals [196–198]. For instance, three-dimensional porous nHA/chitosan (nHA/CS) composite scaffolds, synthesized via a freeze-drying process, demonstrated high porosity, superior swelling behavior and improved mechanical and biological performance compared to chitosan alone [199]. Furthermore, it was found in 2022 that the incorporation of nHA and collagen into the alginate (Alg) microcapsule hydrogel reduced swelling and degradation ratios while increasing compressive strength, enhancing the osteogenic potential of MG63 cells [200]. Notably, the concentration of nHA within these composites significantly influences their physicochemical properties and biological responses. An Alg-based hydrogel with a 30% nHA content exhibited enhanced osteoblastic cell proliferation and activation *in vitro*, along with improved collagenous deposition and trabecular bone formation *in vivo* [201]. However, higher concentrations of nHA (50% and 70%) were found to impair the biological response.

#### Bioceramic scaffolds with nHA

Bioceramics are classified according to their interaction and fusion with bone, often distinguished as bioinert ceramics (e.g. silicon carbides, alumina, zirconia, pyrolytic carbon) and bioactive ceramics (e.g. hydroxyapatite, bioglass, calcium silicate) [202–204]. Extensive research has demonstrated that bioceramics with proper chemical composition and structure not only exhibit osteoconductivity but also possess osteoinductivity, namely, the ability to induce ectopic bone formation in non-osseous sites [205, 206]. Bioceramics promote osteogenesis through their surfaces, which absorb osteoinductive factors like bone morphogenetic proteins and release essential ions such as calcium and phosphate, essential for cell proliferation and differentiation [207]. To augment the properties of bioceramics, various strategies have been employed, including bioactive coatings, physical modifications, and the development of composites with metals and polymers [167, 194, 208]. Incorporating bioinert ceramics like alumina and zirconia has significantly enhanced the mechanical properties of these coatings [209]. Meanwhile, research into integrating nHA with bioinert ceramics to boost their biological activity, though limited, has yielded promising outcomes [210]. Methods such as mixing, impregnation, and vacuum infusion have been used to integrate nHA into bioceramics, enhancing their osteogenic properties. Modal *et al.* [211] synthesized granular nHA and bioactive glass ceramics using co-precipitation and ultrasound-assisted sol-gel methods, demonstrating enhanced cell attachment and proliferation of the MG-63 osteoblast-like cell line. Bioactive fibers composed of nHA-grafted E-glass (nHA/E-glass) were synthesized using the microwave irradiation technique [212]. The incorporation of nHA/E-glass fibers at different weight percentages (0–60%) compromised resin-filler adhesion and affected the water sorption behavior of the composite material. A biphasic calcium phosphate (BCP) substrate was fabricated using the sponge replication method and coated with a uniform layer of nHA [213]. The scaffolds facilitated osteogenic differentiation and induced ectopic bone formation when implanted into the dorsal muscle of rabbits for 90 days.

The surface microstructure of bioceramics plays a pivotal role in bone regeneration, with recent advancements demonstrating that nHA layers constructed *in situ* on bioceramic scaffolds can significantly enhance their biological performance. In one study, a micro/nano-rod hybrid hydroxyapatite surface was fabricated on a 3D-printed scaffold through a hydrothermal treatment process involving a dissolution-precipitation stage [214]. This process resulted in a biomimetic hierarchical structure, featuring interconnected porous composite bioceramics with an overlay of micro/nano-rod hybrid hydroxyapatite. The surface layer facilitated cell adhesion and upregulated cellular differentiation *in vitro*. Further, *in vivo* studies demonstrated its efficacy in promoting capillary formation, bone augmentation, and new bone matrix formation. In another study, a 3D-printed interconnected porous bioceramics β-tricalcium phosphate/collagen scaffold was fabricated, and a biomimetic hydroxyapatite apatite coating was developed *in situ* through a hydrothermal reaction [215]. This coating significantly enhanced cell proliferation, ALP activity and the expression of osteogenic genes. *In vivo* evaluations confirmed that the hydroxyapatite-coated scaffolds promoted bone formation and strengthened bone quality. Researchers also achieved precise control over the micro/nano-structures on the surface of hydroxyapatite scaffolds by modulating Cu^2+^ concentrations during the hydrothermal treatment [216]. *In vitro* endothelial cell cultures demonstrated significantly enhanced cell proliferation. *In vivo* tests conducted on New Zealand rabbits showed that the scaffold with the flower-like surface stimulated significantly more angiogenesis compared to the control scaffold.

### Potential clinical applications of nHA composites

As mentioned above, nHA composites have demonstrated exceptional efficacy in promoting cell proliferation and osteogenic differentiation, positioning them as a cornerstone material for tissue regeneration and repair [217]. In dentistry, nHA composites have found diverse applications, ranging from dental sealants and caries repair to treatments for enamel hypersensitivity and as components of dental implants [218]. Their bioactivity and compatibility with dental tissues make them invaluable in enhancing oral health outcomes. Beyond their regenerative capabilities, nHA composites have also shown promising potential in cancer treatment. Their ability to inhibit tumor growth and support targeted therapy positions nHA as a novel agent in oncology, with modifications such as magnetic nHA paving the way for precise tumor detection and targeted anti-tumor strategies. The clinical efficacy and functionality of nHA composites are significantly influenced by their composition, surface morphology and mechanical properties. These factors are crucial in determining the composites’ drug-loading capacities, release kinetics, cellular interactions and overall biological stability, ultimately affecting their performance in medical applications (Table 3) [220]. Given their versatile nature, nHA composites hold the potential to revolutionize drug delivery systems and cancer therapy strategies, signifying a paradigm shift in personalized and targeted medical treatments.

**Table 3.** Physicochemical properties of various nHA composites

Scaffold materials	Method	nHA shape	nHA Size	Porous structure	Mechanical properties	Outcome	Ref.
Ti-6Al-4V	Atmospheric plasma spraying with hydrothermal treatment	Rod	35 nm	/	/	Promoted adhesion, proliferation, differentiation of MSCs, improving osseointegration between the implants and the surrounding bone tissue	[168]
Zn	Microwave-assisted method	Rod, plate, flower	/	The coating was ∼ 14 ± 2 µm	/	Exhibited high corrosion resistance and offered enhanced antibacterial activity against *S. aureus*	[176]
PEEK	Sonochemical technique	Rod	9, 16 nm	Pore size: 104 ± 34, 146 ± 38 nm, coating thickness in the range of 200–760 nm	/	Enabled a comparatively large amount of drug loading per unit area	[185]
CS	Freeze-drying	Rod, spindle	30–60, 40 nm	Porosity of 78–85%, pore size 50–150 μm	Young’s modulus of 37.26 ± 1.00, 27.37 ± 0.36 kPa	High mechanical properties, improved cell attachment, and osteogenic differentiation	[199]
PLA	Fused deposition modeling	Rod	50 × 80 nm	Pore size（300∼400 μm）	Compressive strength of 17.80 MPa	Enhanced cell adhesion and growth, promoted bone conduction and osteoinduction	[192]
Hydrogel	Direct mixing	/	<200 nm	Pore size（100∼200 μm）	/	Promoted alveolar bone regeneration without the addition of growth factors	[198]
BCP	Vacuum infusion	Rod	55 nm	The coating was ∼ 1 µm	/	Favored cell adhesion and promoted osteogenic differentiation of MSCs and induced ectopic bone formation *in vivo*	[213]
Gelatin	Freeze-drying	Needle	(122 ± 23) × (21 ± 4) nm	Pore size: 121 ± 12 μm, 190 ± 26 μm	Compressive strength of 24 ± 1, 50 ± 3, 17 ± 4 MPa	Supported the attachment, proliferation, and in-growth of cells	[219]

#### Bone repair with nHA composites

The interactions between biomaterials and the host body can elicit various responses, such as inflammation, granulation tissue development, foreign body reactions and fibrous capsule development, depending on the physicochemical and biological properties of the implants [221, 222]. In bone repair, significant research has focused on improving the surface properties of biomaterials to enhance osseointegration, ensuring a robust implant–tissue interface and promoting bone formation. The incorporation of nHA into various materials has shown promising outcomes in promoting osteogenesis, angiogenesis and overall bone regeneration [201, 202]. For instance, a nanotube array with microsphere-like nHA crystals on titanium dioxide nanotube arrays demonstrated enhanced osteoblast differentiation and local factor production [223]. Porous nHA/polyamide 66 (PA66) struts significantly enhanced intervertebral bony fusion in a goat cervical discectomy model [224]. In 2023, an injectable nHA/type I collagen paste was invented and found to promote cell attachment, proliferation, osteogenic expression and tendon–bone interface regeneration *in vivo* [225]. Furthermore, microfluidic technology was utilized to prepare nHA/chitosan composite microspheres, which showed excellent biocompatibility and effectively promoted bone regeneration in cranial defects of rats [226]. The combination of magnetic field treatment and nHA coating significantly improved fusion rates, bone growth and mechanical stability in posterolateral lumbar fusion procedures [227]. A silk fibroin-based coating with a collagen-like structure, embedded with nHA and silver nanoparticles, exhibited enhanced antibacterial and osteogenic abilities [228]. These findings highlight the potential of nHA-based materials in influencing various aspects of bone repair, from cellular responses to overall tissue regeneration.

**Figure 6.**
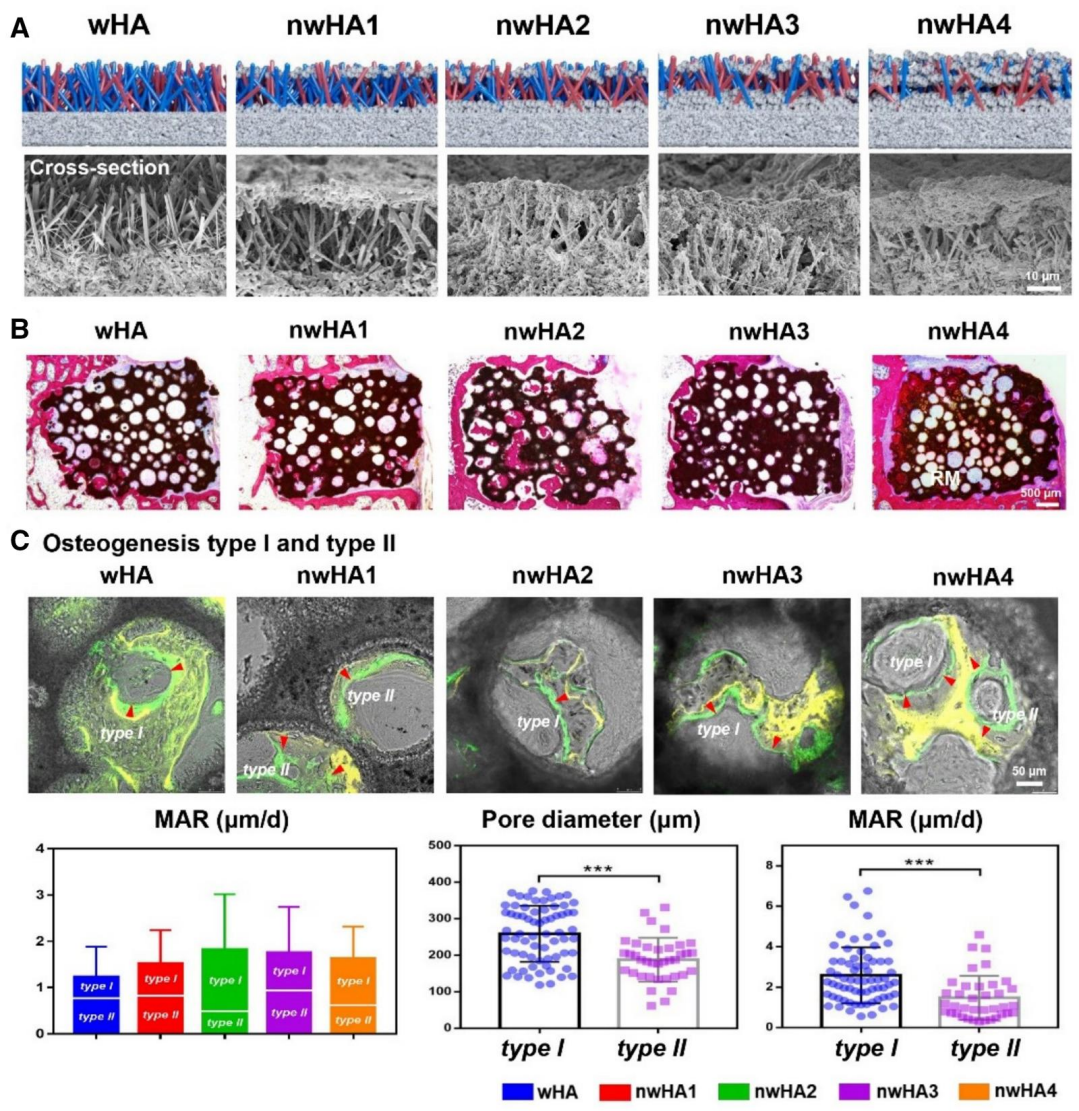
*In vivo* evaluation of bone healing effect of nHA loaded micro-whiskered hydroxyapatite (nwHA) bioceramics implanted in an osteoporotic rat model with bone defect. (**A**) Schematic diagram and SEM morphology of micro-nanostructured hydroxyapatite (nwHA) bioceramics. (**B**) Histological staining analysis and biomechanical test for osteoporotic bone regeneration induced by nwHA bioceramics. (**C**) Sequential fluorescence labeling of new bone formation inside nwHA bioceramics, with tetracycline and calcein labels shown in distinct fluorescence. Osteogenesis modes include type I osteogenesis(the direction of new bone formation is toward the adjacent hole wall) and type II osteogenesis (the direction of new bone formation is away from adjacent pore walls); comparison of mineral apposition rate (MAR) between different nwHA groups; statistical analysis of the relationship between osteogenesis type and pore diameter of the bioceramics, and the relationship between osteogenesis type and the MAR [234]. Copyright 2023, Elsevier.

Osteoporosis, characterized by reduced bone mineral density and compromised bone tissue microstructure, leads to increased bone fragility and fracture risk due to an imbalance between bone formation and resorption [184]. nHA has shown promise in osteoporosis treatment, as it closely resembles the smaller crystal size and lower crystallinity of hydroxyapatite in osteoporotic bone [229]. Studies have demonstrated the positive impact of nHA composites on osteoporotic bone regeneration [230]. Co-culturing osteoblasts derived from osteoporotic rat bones with nHA particles resulted in significant enhancements in osteoblast proliferation and differentiation [61]. Innovations in nHA-loaded materials, including polymers like PEKK and poly(epsilon-lysine), have demonstrated their efficacy in promoting MSCs attachment, proliferation and osteogenic differentiation, thereby facilitating osseointegration [184, 231, 232]. Such advancements are crucial in the context of osteoporosis treatment. Li *et al.* [233] developed hydroxyapatite-coated superparamagnetic iron oxide nanoparticles (SPIO@HA) that target both osteoclastogenesis and osteogenesis, with an optimal Fe/Ca ratio of 1:15. This composition not only significantly reduced bone loss in ovariectomized mice, enhancing bone mineral density by 9.4%, but also showed high bone accumulation and a good safety profile, regulating osteoclast differentiation via the TRAF6−p62−CYLD signaling complex. Meanwhile, hydroxyapatite bioceramics with releasable nHA layers demonstrated enhanced osteogenic differentiation, promoting bone regeneration and vascularization when implanted into critical bone defects in osteoporotic rats (Figure 6) [234].

#### nHA composites in dental applications

nHA composites have been widely used in various dental procedures, contributing to teeth whitening, enamel surface polishing pastes, dental implant coatings, dental fillings, anti-sensitivity agents and caries prevention [235]. For instance, incorporating 5% or 10% nHA into a resin infiltration agent effectively enhanced artificial enamel caries surfaces by improving their smoothness, mineral density and resin penetration [236]. Despite challenges posed by the protein layer influenced by the acquired film formed by oral conditions, the bond between nHA and dental material surfaces remains intact, even in the presence of the film [65, 237]. Enamel demineralization, often manifested as ‘white’ spots, can be effectively treated with nHA, leveraging its remineralization potential to restore enamel integrity and prevent further decay. Studies indicated that nHA enhanced tooth remineralization by stabilizing pH and reducing the presence of *Streptococcus mutans* [238]. Furthermore, nHA infiltrated the micropores in early caries lesions, acting as crystal nuclei in the remineralization process [239]. It attracted calcium and phosphate ions from oral fluids, promoting natural remineralization, enhancing crystal integrity and supporting growth. For example, toothpaste containing 5% nHA effectively facilitated the remineralization of initial cavities and prevented demineralization of healthy enamel [240]. Another development involved the creation of a dental filling material (NDRM) using nHA, gelatin and acrylic acid [241]. This material demonstrated excellent resilience against acidic and basic environments, as well as hot and cold conditions.

Dentin hypersensitivity (DHS), characterized by tooth soreness due to exposure to external stimuli, often results from damaged enamel integrity and exposed dentin [242, 243]. nHA has shown promise in addressing DHS by forming a protective layer on dentin surfaces, effectively sealing dentinal tubules with mineral hydroxyapatite, and reducing dentin permeability [244]. For instance, a paste composed of γ-polyglutamic acid (γ-PGA) and nHA was developed, rehardening tooth microhardness and providing protection against acid etching [245]. Moreover, nHA composites have demonstrated the ability to stimulate dentin formation by promoting the differentiation of dental pulp stem cells (DPSCs) [246]. Combination eggshell-derived nHA (20–50 nm) and carboxymethyl chitosan positive affected cell viability and proliferation while upregulating the expression of dentine sialophosphoprotein (DSPP) and vascular endothelial growth factor (VEGF) in cultured DPSCs [247]. In dental adhesive research, incorporating nHA at various concentrations induced odontoblast differentiation of DPSCs, leading to dense reparative dentin formation in a rat dental pulp exposure model [248]. Furthermore, PCL nanofibrous scaffolds incorporated with nHA facilitated adhesion, spreading and odontogenic differentiation of DPSCs [249]. Bioresorbable antibacterial PCL-PLA-nHA composite membranes designed for oral and maxillofacial defects exhibited bactericidal properties while promoting osteogenesis, making them suitable for dental and maxillofacial surgery [250]. Additionally, nHA mineralized silk fibroin (MSF) scaffolds induced osteogenic differentiation in MC3T3-E1 cells *in vitro* and promoted new bone regeneration within tooth extraction sockets *in vivo*, addressing alveolar ridge resorption following natural tooth loss [251].

#### Cancer treatment with nHA composites

nHA composites have emerged as a promising strategy for addressing the multifaceted challenges of tumor-induced damaged tissues. These composites not only induce apoptosis in tumor cells but also stimulate cellular infiltration and enhance the healing process, offering a comprehensive therapeutic approach where traditional methods may be insufficient [13, 134]. For instance, Zhang *et al.* [90] conducted a study involving the fabrication of porous Ti scaffolds coated with nHA, which were subsequently implanted into segmental bone defects in tumor-bearing rabbits. The controlled release of nHA not only significantly inhibited tumor growth but also facilitated bone regeneration. Similarly, Shao *et al.* [252] developed a porous Mg-doped wollastonite bioceramic scaffold coated with nHA, demonstrating its efficacy in slowing scaffold degradation, inducing apoptosis in osteosarcoma cells, and promoting osteogenic differentiation of MSCs. Furthermore, Ma *et al.* [253] investigated the effect of nHA/graphene oxide (nHA/GO) composite particles with different proportions on human osteosarcoma cells (HOS), pre-osteoblastic MC3T3-E1 cells and MSCs with or without 808-nm NIR light irradiation. The scaffolds exhibited dual functionality, effectively eradicating HOS under 808-nm NIR irradiation and promoting osteogenesis in MSCs. Such scaffolds further demonstrated superior post-operative bone formation in cranial defects of rats, indicating its potential in enhancing bone regeneration and combating osteosarcoma. Additionally, to address post-operative skin defects and prevent cutaneous melanoma (CMM) recurrence, Xu *et al.* [254] developed a chitosan/alginate (CS/Alg) hydrogel infused with nHA. This novel hydrogel demonstrated effective tumor growth inhibition through mitochondrial-dependent apoptosis and showed no biosafety risks.

nHA composites have also shown significant potential in the fields of early detection and targeted molecular therapy. For example, a nano-theranostic system was developed in 2022 by combining iron and hydroxyapatite materials, resulting in Fe_3_O_4_-HAp magnetic hydroxyapatite nanocomposites (MHNCs) through a straightforward solvothermal synthesis [255]. When combined with indocyanine green (ICG), the MHNCs demonstrated strong absorption in the NIR region and generated significant heat upon laser irradiation, leading to the apoptosis of cancerous cells. In another study, PEEK scaffolds modified with molybdenum disulfide (MoS_2_) nanosheets and nHA exhibited dual-effect synergy, demonstrating photothermal therapeutic (PTT) properties upon NIR irradiation [256]. The modified scaffolds reduced the viability of osteosarcoma cells *in vitro*, and the incorporation of nHA enhanced the proliferation and adhesion of bone cells. Additionally, Cheng *et al.* [257] introduced an organic–inorganic hybrid nanosystem comprising poly(acrylic acid) (PAA), nHA and ICG, tailored with glucose for targeted delivery. This nanosystem exhibited enhanced cellular uptake, resulting in increased intracellular concentrations of ICG and Ca^2+^ through glucose transporter 1 (GLUT1)-mediated endocytosis. The excessive Ca^2+^ induced cell or organelle damage, while ICG triggered photothermal and photodynamic effects (PTT/PDT) upon laser irradiation, intensifying cell toxicity and apoptosis. *In vivo* assessments demonstrated good hemocompatibility and biosafety, with enhanced drug accumulation in tumor tissues leading to a significant tumor growth inhibition rate.

### Therapeutic delivery via nHA composites

One critical challenge in systemic drug delivery is the rapid clearance of drugs by the liver and spleen, leading to poor bioavailability and suboptimal therapeutic outcomes [258]. The unique properties of nanomaterials enable precise control over drug release, enhanced drug stability, improved bioavailability, and targeted delivery to targeted delivery to specific tissues or organs [259]. The nHA composites facilitate the encapsulation and controlled release of a wide range of therapeutic molecules, from small pharmaceuticals to large biomacromolecules, tailoring to the diverse needs of medical treatments (Table 4). nHA composites, through their engineered surface properties and targeted delivery capabilities, can potentially reduce rapid clearance, enhancing the drug's bioavailability [269, 270]. However, developing nHA delivery systems faces challenges such as rapid drug clearance, degradation and uncontrolled release at the target site. Addressing these issues requires innovations in nanoparticle engineering, surface modification and controlled release formulations to unlock the full potential of nHA-based delivery systems. Furthermore, the encapsulation of stem cells within these nanostructured materials opens new avenues in regenerative medicine, aiming to harness the inherent regenerative capabilities of stem cells for tissue engineering.

**Table 4.** Representative recent studies using nHA nanocomposites for drug and bioactive substance delivery

Drug/factor	Scaffold	Method	Load capacity	Release profile	Outcome	Refs
PTH(1–34)	CS/nHA	Direct mixing	10–50 μg/ml	A constant release of PTH(1–34) over 10 days	Enhanced osteogenic differentiation of MSCs, leading to efficient bone regeneration in a rat cranial defect model	[260]
Cal	PDLLA-PEG/nHA	Freeze drying	/	Gel alone showed a high initial burst release of up to 80% within the first 1–2 days. Gel+HA and Gel+HA-D exhibited a lower initial burst, with continuous Cal release observed over 35 days	Promoted osteogenic differentiation of MSCs, and *in vivo* studies verified that the composite Cal-loaded system enhances bone regeneration in critical-size femoral epicondyle bone defects	[261]
Vancomycin	Gelatin/nHA/PLLA	Entrapment or absorption method	Antibiotic absorbed groups (>50%) exhibited better encapsulation efficiency than entrapped groups (<30%)	Demonstrated sustained delivery of vancomycin, exhibiting bactericidal properties for 30 days	Eliminated bacteria and promoted new bone formation within 3 months	[262]
EGCG	nHA	Template method	100 μmol·g^−1^	The release rate of EGCG was promoted at pH 6.5 and in the presence of papain, with over 56% of EGCG released at 72 h	Promoted osteogenic differentiation and exhibited superior anti-osteosarcoma effects both *in vitro* and *in vivo*	[263]
BTZ	SA/nHA	Direct mixing	The encapsulation rate of BTZ was about 77.62%	The total mass reached 74.7% (pH 7.4) and 74.4% (pH 6.5) of the initial weight at 24 days	Exhibited dual functions of inhibiting tumor recurrence and promoting bone tissue regeneration simultaneously	[264]
TXA	SA/nHA	Direct mixing	91 ± 0.4% ∼ 94 ± 0.7%	More than 50% of TXA was released within the first 30 min, followed by a sustained phase, releasing over 95% within 2 h	The clotting time and recalcification time was reduced by 69% and 80%, respectively	[265]
BMP-2 and VEGF	SF/nHA	Chemical and physical adsorption	/	An initial burst release of 6.7% of BMP-2 was observed on the first day, slowing to approximately 15.5% after 28 days. VEGF showed a rapid initial burst of 48.7% on the first day, with 66.4% released in a sustained manner within the first 10 days	The rapid initial release of VEGF promoted angiogenesis, while the slow and sustained release of BMP-2 facilitated osteogenic differentiation. The combination of BMP-2 and VEGF produced a synergistic effect on bone formation, surpassing the effects of BMP-2 or VEGF alone	[266]
Gentamicin sulfate	PU/nHA	Solvent casting and the evaporation method	/	A sustained release profile was observed in all samples, with cumulative GES release ranging from 83% for PU 5 GES to 98% for PU 10 HA GES	All composites showed antibacterial activity against *S. epidermis* and *E. coli strains* over 3 days in the agar-diffusion assay	[267]
Tofacitinib	nHA-GLT	Physical adsorption	Drug entrapment efficiency of 90.7%	Tofa-nHA-GLT NPs exhibited 86.26 ± 2.9% release in a sustained pattern, and Tofa-nHA-GLT NPs gel showed 69.25 ± 1.8% sustained drug release within 72 h	The sustained drug release profile at pH 6.5 (arthritic joint pH) and increased cellular uptake of the dye-loaded formulation compared to a plain dye solution augmented localized delivery at inflamed sites	[268]

#### nHA composites for drug and bioactive substance delivery in bone repair

In orthopedics, nHA stands out as a versatile carrier for delivering a wide range of drugs, cells, and bioactive molecules, enabling targeted delivery to bones and controlled release of specific drug [261, 262, 271, 272]. For example, a composite hydrogel incorporating parathyroid hormone (PTH) peptide PTH (1–34) and nHA demonstrated the ability to promote osteogenic differentiation of MSCs through Notch signaling *in vitro* [260]. Implantation into a rat skull defect model showed synergy between nHA and PTH, facilitating bone regeneration. In another study, insulin-loaded PLGA nanospheres were integrated into nHA/collagen (nHAC) scaffolds [273]. *In vitro* experiments showed favorable biological functions of the insulin-loaded nHAC/PLGA composite scaffolds, including enhanced adhesion, proliferation, and osteoblast differentiation of MSCs. The composite scaffold exhibited enhanced bone regenerative capacity in a rabbit mandible critical size defect. Furthermore, the versatility of nHA in drug delivery systems is exemplified by its high affinity and anti-absorptive properties toward various drugs. For instance, aminated modified polylactic acid (EPLA) microspheres underwent mineralized crystallization of nHA on modified PLA nanofibrous microspheres, resulting in EPLA/nHA composite microspheres with superior drug loading capacity and sustained release performance for alendronate due to strong chemical bonding interactions [274].

Plant-derived substances, including ginsenoside, polyphenols, and astragalus, possess inherent qualities that stimulate antioxidant activity, angiogenesis, and osteogenesis, thereby facilitating an environment conducive to bone healing and regeneration [275]. The integration of plant-derived substances into nHA delivery systems has shown significant promise in enhancing bone regeneration and promoting angiogenesis [276]. For example, Mohammadpour *et al.* fabricated a nanocomposite hydrogel based on alginate (Alg) and nHA, enriched with phenolic purified extracts from the aerial parts of Linum usitatissimum (LOH) [277]. This hydrogel played a crucial role in mitigating oxidative stress, a common impediment to tissue regeneration, thereby positioning it as an ideal scaffold for bone tissue engineering. In another study conducted in 2022, gum tragacanth (TG)-functionalized nHA (TG-HAp) with needle-shaped nanoparticles was developed [278]. TG-HAp increased cellular VEGF expression in MG-63 cells and demonstrated angiogenic properties in *in vitro* tube formation assays using human umbilical vein endothelial cells (HUVECs), indicating its potential to promote both angiogenesis and osteogenesis. Similarly, surface amino-functionalized nHA was synthesized, and Epigallocatechin-3-gallate (EGCG) was introduced to obtain HA-EGCG [263]. This compound not only exhibited enhanced anticancer properties *in vitro* and *in vivo* but also promoted osteogenic differentiation, illustrating its dual utility in both cancer therapy and bone restoration.

MSCs are considered promising cell sources for bone tissue engineering due to their multi-lineage differentiation potential and extensive self-renewal capacity [279]. The integration of MSCs with nanocomposites has significantly advanced in bone tissue engineering, promoting efficient tissue repair and regeneration [280, 281]. For example, Kulanthaivel *et al.* encapsulated MSCs within a matrix comprising cobalt-doped nHA (HAN), gum tragacanth (GT), and calcium alginate (CA) [282]. This composite promoted osteogenic differentiation in stem cells and induced high levels of hypoxia-inducible factor 1 alpha (HIF-1α) and VEGF expression in MSCs, indicating its potential for angiogenesis and tissue repair. Furthermore, nanocomposites serve as effective carriers for delivering bioactive molecules that simulate the natural expression patterns of essential growth factors during bone repair [283, 284]. For example, a heparin-conjugated strontium-substituted hydroxyapatite/silk fibroin (Sr-nHAp/SF-Hep) scaffold loaded with BMP-2 provided sustained release, enhancing MSC proliferation and osteogenic differentiation, thus improving new bone regeneration *in vivo* [285]. Current research includes efforts to simulate the spatiotemporal expression of growth factors such as BMP-2 and VEGF using silk fibroin (SF)/nHA scaffolds [266]. This approach led to complete bone bridging in rat calvarial defects after 12 weeks.

#### nHA composites as carriers for drugs and bioactive substances in dentistry

Periodontal disease is a common multifactorial, inflammatory, microbiome-related condition characterized by chronic infection and inflammation in periodontal tissues, leading to the destruction of alveolar bone and, ultimately, tooth loss [286]. The objective of periodontal tissue treatment is to restore the physiological function of teeth by rebuilding damaged components, including alveolar bone, periodontal ligament, and cementum, and thereby integrate with the tooth root to restore a functional and robust tooth attachment [287]. After natural tooth loss, the resorption of the alveolar ridge presents significant challenges for dental restoration. Biomaterial scaffolds have emerged as a formidable solution for alveolar ridge preservation post-tooth extraction [288]. For example, Fang *et al.* in 2022 developed a gelatin/nHA/metformin scaffold (GHMS) by co-precipitating calcium hydroxide and orthophosphoric acid within a gelatin solution, incorporating metformin, and cross-linked by microbial transglutaminase [289]. In critical-sized rat alveolar bone defects, GHMS exhibited exceptional alveolar ridge preservation and facilitated enhanced bone formation, surpassing the performance of commercially available xenografts (Bio-Oss collagen) and allogeneic (Sinbone particles). Similarly, Gao *et al.* prepared PLA/nHA scaffolds combined with vancomycin (Van)-based chitosan (CS) hydrogel (Gel@Van) to form a local antibiotic release system (PLA/nHA/CS-Van) [290]. This scaffold provided sustained antibiotic release for over 8 weeks, effectively inhibiting the growth of *S. aureus* without compromising the proliferation and differentiation of MC3T3-E1.

Furthermore, toothpaste containing nHA has demonstrated efficacy in treating dental hypersensitivity after teeth whitening, and the repair of hard tissue damage can be further accelerated through compound drugs or factors [67, 68]. Researchers utilized the sol-gel technique to prepare nHA and then modified it using Elaeagnus extract [291]. The modified particles increased the expression of immunomodulatory/dentin-pulp regeneration genes in DPSCs, including human leukocyte antigen-G5 (HLA-G5), VEGF, DSPP, and interleukin 6 (IL6). This indicated the potential of the modified nHA in enhancing the stemness capability of DPSCs for treating inflamed or damaged pulp. Wang *et al.* used nHA-modified collagen biomimetic material (nHAC) as a carrier for fibroblast growth factor (bFGF) in periodontal tissue regeneration [292]. The scaffold effectively loaded bFGF, providing a conducive environment for human periodontal ligament cells (HPDLCs) to grow and attach. *In vivo* experiments demonstrated the efficacy of the composite membrane, promoting new alveolar bone growth and cement formation, and facilitating the regeneration of periodontal tissue. In addition, natural animal bone-derived nHA was employed as a nonviral gene carrier [293]. When modified with Polyethyleneimine (PEI), nHA exhibited excellent distribution, a strong positive charge, outstanding gene transfer ability, and low toxicity. Delivery of BMP-2 with pEH to DPSCs was effective in promoting bone differentiation, showing the potential of nHA in gene therapy applications for dental tissue regeneration.

#### nHA composites for targeted cancer therapy delivery

nHA composites, featuring large surface area, high drug capacity, and pH-responsive release, effectively encapsulate and deliver chemotherapeutic agents, offering a strategic approach to cancer treatment that minimizes traditional chemotherapy's adverse effects [294, 295]. For example, Chen *et al.* in 2023 developed a bifunctional scaffold (BTZ/nHA@SA) by integrating nHA and sodium alginate (SA) and loading it with bortezomib (BTZ), a proteasome inhibitor used in chemotherapy [264]. This scaffold exhibited a dual function in tumor suppression and bone regeneration, as evidenced by *in vivo* evaluations using mouse tumor and rabbit femoral defect models. Dong *et al.* revealed the synergistic enhancement of doxorubicin (DOX) efficacy by nHA, effectively reversing multidrug resistance (MDR) in breast cancer cells [296]. DOX-loaded nHA significantly reduced the half-maximal inhibitory concentration (IC50) value against MDR cells in comparison to DOX alone. Notably, nHA itself thwarted drug efflux in MDR cells, instigated mitochondrial damage, suppressed ATP synthesis, and triggered sustained mitochondrial calcium overload, culminating in apoptosis specifically in cancer cells, while sparing normal cells. Moreover, the combination of nHA composites with natural anticancer agents and advanced therapeutic modalities represents a promising avenue in cancer treatment [263]. These approaches enhance the therapeutic efficacy and addresses the limitations of conventional treatments. For instance, Martins *et al.* demonstrated that combining the low-level chemotherapy paclitaxel (PTX) with nHA reduced cell viability in breast cancer cells (MCF-7 lineage) [297]. This approach induced apoptotic phenotypes and inhibited survival stimulation. Additionally, Zhai *et al.* engineered 32P-labeled hydroxyapatite by chemical synthesis (32P-HAp) and physical adsorption (32P-doped-HAp) [298]. Chemical synthesis significantly improved radiolabel yield and stability compared to physical adsorption.

The utilization of small interfering ribonucleic acid (siRNA) as an advanced therapeutic modality, along with the construction of co-carrier systems, holds great promise for cancer treatment [299]. Overcoming barriers such as siRNA stability, minimizing non-target effects, and diversifying delivery systems are critical for the advancement of siRNA therapies [300]. In a study targeting Stat3 interference in RM1 prostate cancer cells, plasmid-based Stat3 specific short hairpin RNA (sh-Stat3) was delivered by nHA [301]. nHA facilitated the delivery of sh-Stat3, resulting in decreased RM1 cell viability *in vitro*. Polyethylenimine-modified nHA (HAp-PEI) was designed for targeting the KRAS gene, associated with pancreatic carcinoma (PC) [302]. siRNA of the KRAS gene (siKras) was loaded onto HAp-PEI to create functionalized HAp-PEI nanoparticles (HAp-PEI/siKras), effectively knocking down KRAS gene expression and downregulating the KRAS protein in human PC cells (PANC-1). This approach demonstrated significant anti-PC efficacy *in vitro*, particularly against PANC-1, BXPC-3, and CFPAC-1 cells. Furthermore, Chen *et al.* (2022) encapsulated nHA and granulocyte-macrophage colony-stimulating factor (GM-CSF) in PLGA-PEG-PLGA hydrogel, which attenuated the burst release of GM-CSF, facilitating sustained release at the tumor site [303]. This approach enhanced anti-tumor immunity by boosting CD8 T-cell responses, showing potential in cancer immunotherapy. Additionally, tetrahedral DNA nanostructures (TDNs) conjugated with AS1411 aptamer, targeting nucleolin overexpressed on tumor cell membranes, were employed to construct a mono-dispersed hydroxyapatite-based probe with Gd^3+^ doping (Apt-TDNs-GdHAp) for MR imaging [304]. Apt-TDNs-GdHAp probes demonstrated superior T1-weighted imaging performance compared to Microwave-GdHAp, with enhanced stability and tumor-targeting accessibility.

## Conclusions and future perspectives

Significant advancements have been achieved in nHA research, spanning controlled nanoparticle synthesis, composite optimization, biological interactions elucidation, ion doping techniques and advanced drug delivery systems development. Employing various synthesis techniques enables the synthesis of nHA with tailored functionalities such as antimicrobial activity, improved cell adhesion, precise drug release and superior mechanical properties—critical for nHA’s success in various applications [305]. However, challenges persist in achieving precise control over the size, shape, structure, and functionality of nHA to address diverse biomedical applications. Balancing superior physical and chemical qualities with desired biological functions in a single material presents a significant challenge. nHA composites present a viable solution, offering the flexibility to tailor composition, structure and morphology. This customization facilitates the emulation of natural tissue properties, enhancing the applicability of nHA in various biomedical contexts. In bone tissue engineering, the focus is on addressing mismatched degradation rates with osteogenesis, enhancing biological activity and deepening the understanding of mechanical properties [306]. In dentistry, the integration of nHA into oral care products promotes enamel remineralization by creating ion supersaturation at lesion sites, but faces challenges in direct dentinal tubule applications due to particle size and binding weaknesses [307, 308]. A realistic evaluation of nHA’s functionality in oral environments necessitates considering dynamic intraoral conditions, such as salivary flow and oral microbiota [65]. The anti-tumor potential of nHA is opening new avenues in cancer therapy, aiming for bone defect repair post-tumor excision and potentially diminishing tumor recurrence.

Investigating the biological effects of nHA and its composites is crucial for expanding their clinical applications and establishing a theoretical foundation for innovative biomaterials that integrate repair and treatment. Enhancements in biological functions such as increased bioactivity, antibacterial properties, multimodal imaging capabilities and cancer diagnosis and treatment potential are achievable through strategic functionalization treatments, ion doping, and advanced drug delivery strategies. Recent research has explored multi-ion doping in nHA composites, which alters the material’s physical and chemical properties by affecting crystal aggregation and particle size. The incorporation of different ions into the apatite lattice can have synergistic or conflicting outcomes, adding a layer of complexity to their functionality [142]. The biological performance of these materials is more profoundly influenced by factors such as grain size, surface morphology and composition than by the choice and concentration of dopants [131]. Furthermore, nanocomposites with various morphologies, such as hollow, core-shell and porous structures, can effectively regulate the delivery of drugs, genes and proteins [309]. Functionalization treatments and stimuli-responsive coatings on these structures act as molecular gates, allowing for precise control over the release of therapeutic agents [92]. However, determining optimal drug dosages within nHA composites remains a significant challenge that necessitates further research to achieve targeted therapeutic outcomes. The complexity escalates with multifunctional composites that integrate ions and anticancer drugs, complicating the quantification of their therapeutic impact. Challenges such as bioavailability, targeting efficiency and potential cytotoxicity are inherent in nanocomposite-based systems used for drug and bioactive molecule delivery. While current research focuses predominantly on structural aspects influencing delivery, there is a lack of consensus on *in vivo* release dynamics and cellular internalization.

**Figure 7.**
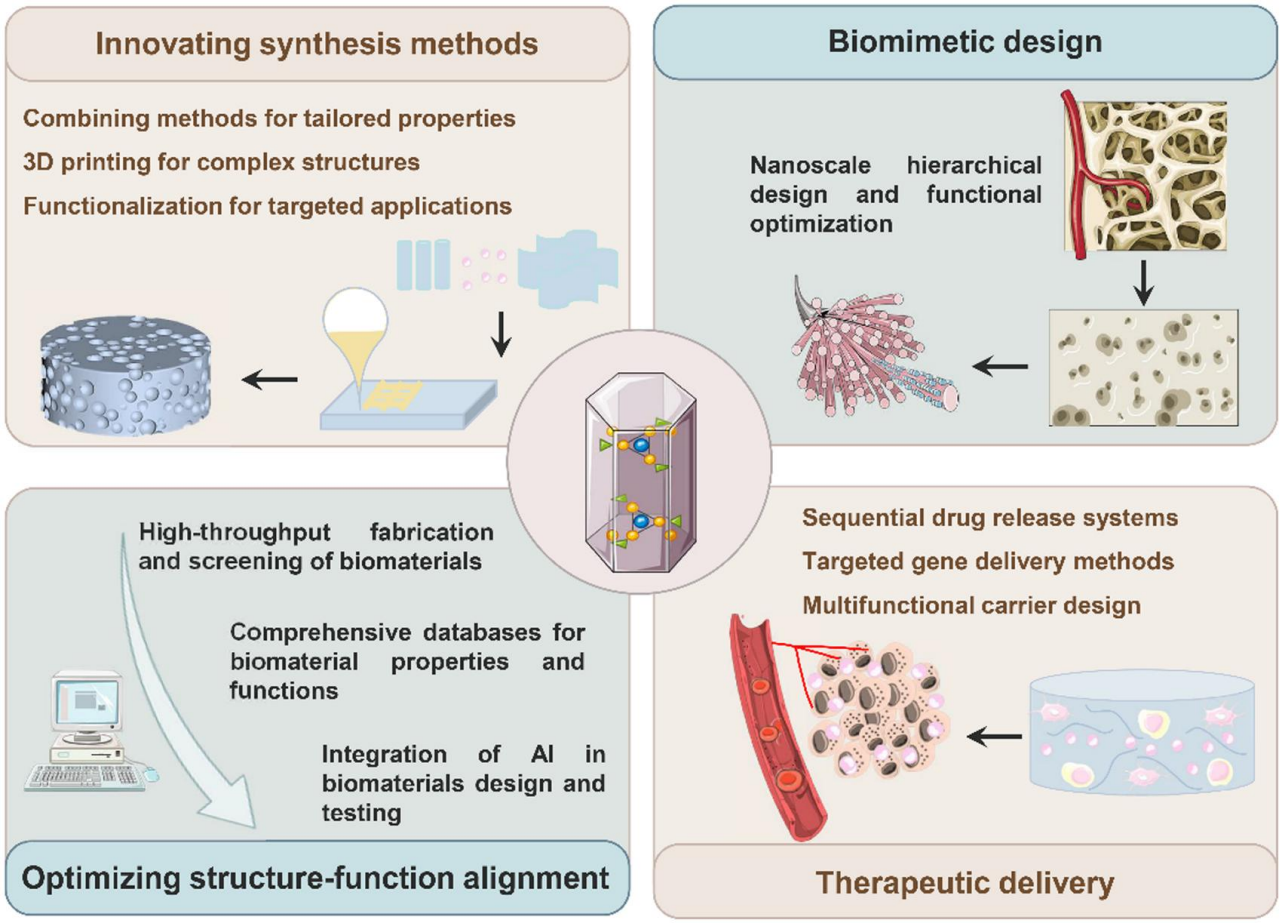
Perspective key issues regarding the development of nHA and composites.

To summarize, key areas listed in Figure 7 require careful consideration for future research on nHA and its composites. These include: (i) Refinement of synthesis methods: The advancement of nHA and composite materials requires a concerted effort to refine and innovate synthesis techniques. Future research should explore new methodologies integrating polymers, metals and bioceramics with nanoparticles, thereby expanding the material science frontier and catalyzing novel clinical applications in nanotherapeutics; (ii) Understanding mechanisms of action: Delving into the complex interactions between nHA composites and biological systems is paramount for designing materials that can effectively interact with biological tissues and cells. Gaining a thorough understanding of these mechanisms, especially in tissue engineering and drug delivery, will bridge the gap between theoretical insights and their practical biomedical applications; (iii) Biomimetic fabrication: Mimicking the natural architecture of bone and enamel remains a formidable challenge in biomaterials research. Embracing novel mineralization-directing biomolecules and exploring unique mineralization processes could provide pathways to achieving this goal, offering highly oriented, structurally precise biomaterials; (iv) Drug delivery and therapeutic effects: Enhancing the efficacy of drug delivery systems through the functionalization of nHA composites is pivotal. This approach holds the promise of improving treatment outcomes, patient experiences and minimizing adverse effects, thus playing a vital role in the evolution of nanotherapeutics; (v) Exploration of new applications: there is a vast potential for nHA in emerging fields such as responsive biomaterials in immunomodulation, smart drug delivery systems, innovations in beauty products and dynamic surgical environments. In summary, it is essential to conduct a comprehensive analysis of current experimental data and determine the optimal conditions for nHA composites tailored to various medical applications.
